# A Re-examination of Dichoptic Tone Mapping

**DOI:** 10.1145/3443702

**Published:** 2021-04-21

**Authors:** MINQI WANG, EMILY A. COOPER

**Affiliations:** University of California, Berkeley, USA

**Keywords:** Binocular vision, high dynamic range

## Abstract

Dichoptic tone mapping methods aim to leverage stereoscopic displays to increase visual detail and contrast in images and videos. These methods, which have been called both *binocular tone mapping* and *dichoptic contrast enhancement*, selectively emphasize contrast differently in the two eyes’ views. The visual system integrates these contrast differences into a unified percept, which is theorized to contain more contrast overall than each eye’s view on its own. As stereoscopic displays become increasingly common for augmented and virtual reality (AR/VR), dichoptic tone mapping is an appealing technique for imaging pipelines. We sought to examine whether a standard photographic technique, exposure bracketing, could be modified to enhance contrast similarly to dichoptic tone mapping. While assessing the efficacy of this technique with user studies, we also re-evaluated existing dichoptic tone mapping methods. Across several user studies; however, we did not find evidence that either dichoptic tone mapping or dichoptic exposures consistently increased subjective image preferences. We also did not observe improvements in subjective or objective measures of detail visibility. We did find evidence that dichoptic methods enhanced subjective 3D impressions. Here, we present these results and evaluate the potential contributions and current limitations of dichoptic methods for applications in stereoscopic displays.

## 1 INTRODUCTION

A good digital reproduction recreates the visual experience of a real scene using a different medium (e.g., a television, computer monitor, or head-mounted display). The reproduction process is primarily mediated by two devices: a camera and a display. Current pipelines face a number of challenges, particularly related to presenting the full range of visible light in natural scenes [7]. Two common challenges are the reproduction of the absolute range (lowest and highest values) and the reproduction of visually distinguishable differences in light intensity within that range. While both of these factors are perceptually relevant, the latter is more important for creating a reproduction with good fidelity of visual details.

Typical digital reproductions often lack visible details in the highlights or low-lights (shadows) of a scene. This loss of detail occurs because the dynamic range of typical cameras—their ability to represent visually distinguishable light intensities—cannot fully capture the range of detail that the human eye can see. High-end cameras can now represent a wider dynamic range directly; however, these cameras are not broadly available [19]. Instead, various methods exist to synthesize high dynamic range (HDR) images from low dynamic range (LDR) camera images, commonly by computationally combining multiple exposures [1, 4, 6, 28].

Importantly, even when HDR images can be generated, scene reproduction is still limited by the light levels that a display can produce. A tone mapping algorithm is typically used to define a function that maps values from HDR space to an LDR space appropriate for the display. However, this process is lossy and can introduce artifacts [7]. For example, traditional compressive tone mapping curves preserve visible contrast across a certain range of light levels at the expense of reducing contrast in other ranges. Local tone mapping algorithms preserve contrast in fine details, at the expense of reducing global luminance differences across regions of a scene (e.g., References [12, 13, 32]). At present, there is no practical way to faithfully reproduce the full range of visible light intensities in natural scenes with typical devices.

**Fig. 1.**
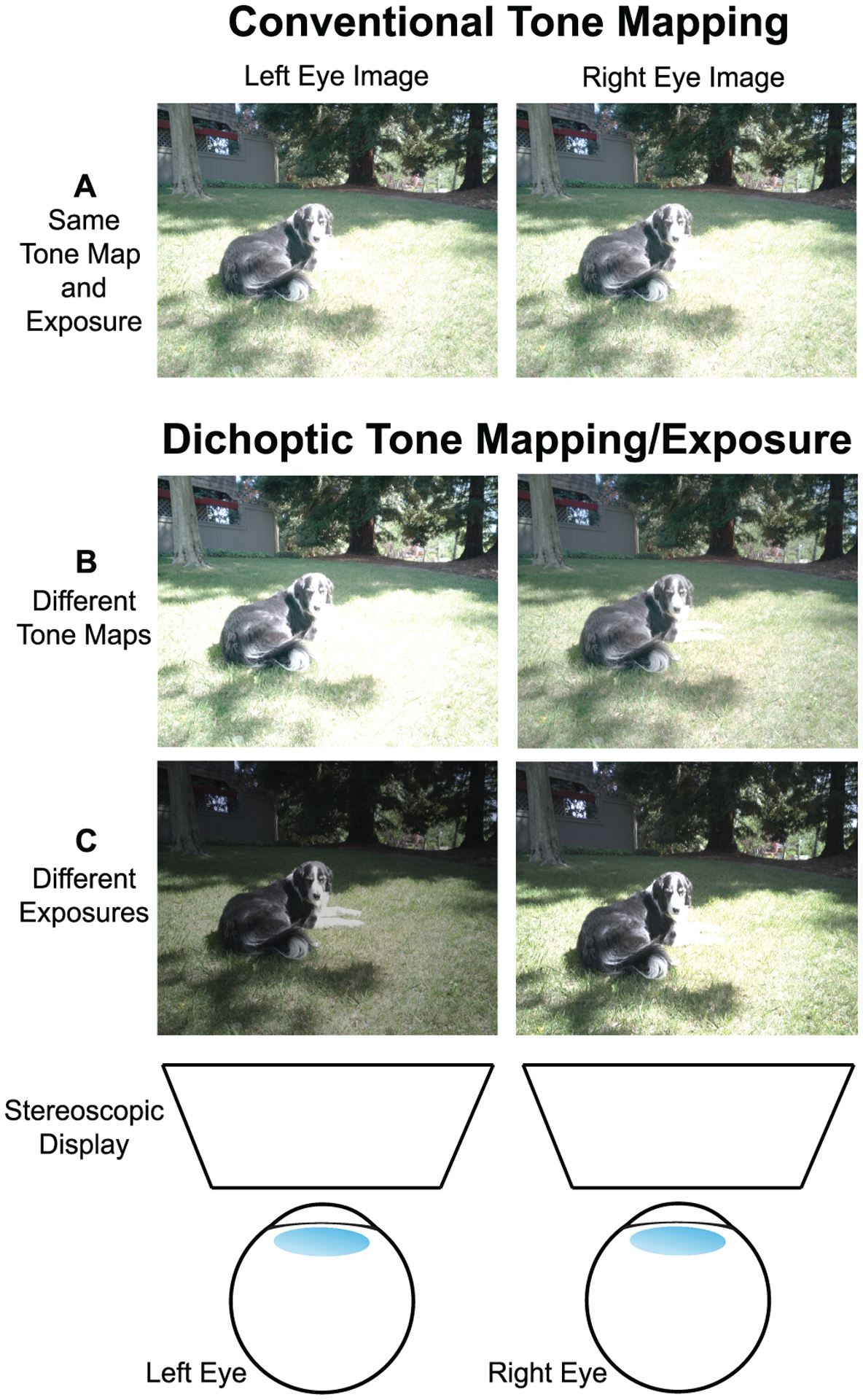
Example images shown to each eye on a stereoscopic display using either conventional digital reproduction methods (A) or dichoptic tone mapping/exposure methods (B, C). (A) In conventional methods, both eyes view images that were produced with the same tone mapping algorithm and camera settings (usually captured from different viewpoints to create binocular disparities). With dichoptic methods, the eyes see images that have different tone maps, emphasizing different areas of detail/contrast in the two eyes (B), or images that have the same tone map, but that were captured with different camera exposures (C). These examples were derived from the HDR+ Burst Photography Dataset [18], and panels A and B use tone maps from Reference [41], reproduced with the authors’ permission.

With the rise of stereoscopic displays to support virtual reality (VR) experiences, a range of new approaches have been proposed that aim to leverage binocular vision to increase visible contrast and detail [14, 37, 40–42]. These approaches use different tone mapping algorithms for the images shown to the left and right eye. The term *dichoptic* refers to situations in which the two eyes are presented with different images, so we will use *dichoptic tone mapping* to refer to these techniques. We will refer to the two different images that make up a given dichoptically tone mapped pair as the *component images*. Examples of a conventionally versus dichoptically tone mapped image pair are shown in Figures 1(A) and (B). If the increased overall contrast from dichoptic tone mapping can be integrated effectively by the visual system, these approaches are appealing for use in VR systems. Motivated by this idea, we sought to extend existing dichoptic tone mapping algorithms with a simple dichoptic exposure method inspired by photography (Figure 1(C)). However, in the process of conducting a set of user studies, we were unable to find evidence that dichoptic tone mapping methods [41, 42] (selected as prior state-of-the-art) or the proposed exposure-based method consistently improved overall subjective image quality or detail visibility. We did observe a substantial enhancement in subjective 3D impressions [42]. These results are important to consider when evaluating how to capture and tone map imagery for stereoscopic displays, and highlight the challenges and opportunities associated with designing dichoptic methods that consistently improve upon conventional imaging approaches.

Our work makes three primary contributions:

1. We re-evaluate previous dichoptic tone mapping methods, along with dichoptic exposures, in a series of six user studies.
2. We provide a new guideline for assessing dichoptic tone mapping, whereby the dichoptic pairs should be compared to each of the component images.
3. We introduce a performance-based perceptual measure for quantifying detail visibility in images to complement subjective metrics used in typical user studies.

## 2 RELATED WORK

### 2.1 Binocular Combination

During normal experience, the visual system is tasked with processing slightly different views in the two eyes, which must be combined to generate a unified percept of the world. The binocular differences encountered during normal experience primarily manifest as *binocular disparities*: slight offsets between features in the two eyes’ images that occur due to the horizontal displacement between the eyes. These binocular disparities are used by the visual system to infer the relative depths of objects and surfaces in the environment [21]. Stereoscopic displays show different images to the two eyes, creating binocular disparities and a compelling three-dimensional (3D) percept.

The binocular differences created by dichoptic tone mapping are different from binocular disparities: The two tone maps create images that differ in luminance, color, and/or contrast, rather than viewpoint. This manipulation can be presented alone (i.e., the eyes see the same scene view but with different tone maps) or in addition to binocular disparity (i.e., the two eyes see different views of the scene and different tone maps).

**Fig. 2.**
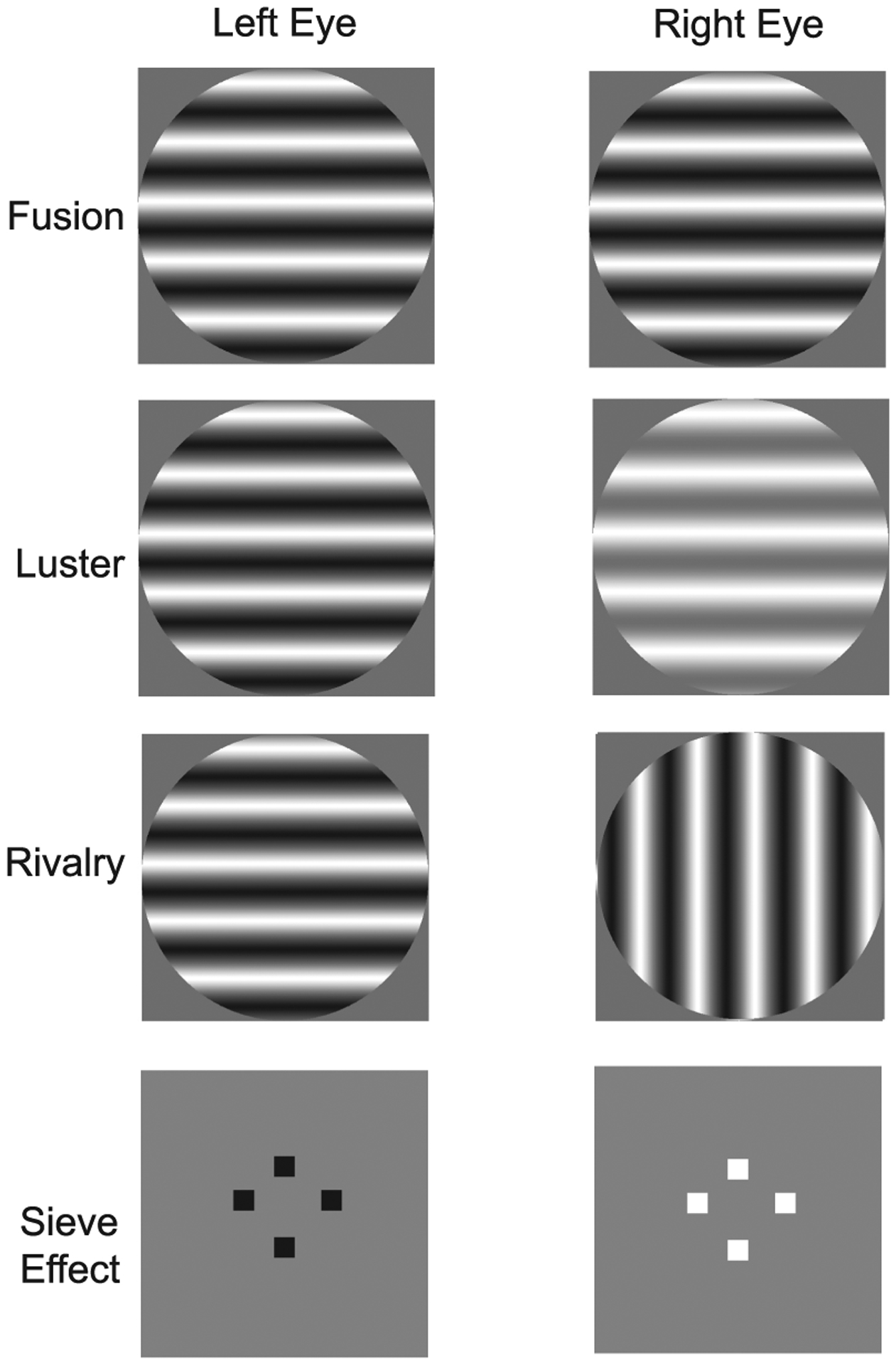
Examples of simple images used to study binocular visual combination. The left and right columns illustrate images shown to the left and right eyes, respectively. These stimuli provide illustrative examples of those that elicit fusion (top), luster (top middle), rivalry (bottom middle), and the sieve effect (bottom). Images may be either divergently fused or cross fused.

Basic vision research suggests that introducing a luminance/contrast difference between the two eyes’ images can elicit a range of perceptual phenomena. When the two eyes view images that are similar in appearance, the visual system is said to “fuse” the images into a single, unified percept (Figure 2, top). Extensive psychophysical studies using simple, synthetic stimuli have shown that fusion can be achieved even when the two eyes’ images differ in luminance or contrast [5, 26, 27]. However, the fused percept is not typically well-described as a simple average of the two eye’s images. For example, studies in which sine wave gratings of different contrast were presented to each eye showed that the binocularly fused percept is generally dominated by the eye seeing higher contrast [22, 26]. If this “winner-take-all” phenomenon is applicable to viewing more complex natural images, fusion may be exploited by dichoptic tone mapping to produce a binocular percept of greater overall contrast than either of the component images. For example, if each component image has some regions with more visible contrast than others, the binocularly fused percept may combine the higher contrast regions of both components to better convey the visual details of the physical scene.

However, viewing mismatched images in the two eyes can also result in a range of secondary perceptual effects. Two such effects are luster and binocular rivalry. *Luster* tends to occur when the overall pattern seen by the two eyes is relatively similar but the luminance, contrast, or color differs [15, 35] (Figure 2, top middle). The term luster refers to the fact that stimuli with this property often appear shimmery, glossy, or metallic. *Binocular rivalry* tends to occur when the eyes view two geometrically dissimilar patterns [27]. For example, if one eye sees a horizontally oriented sine wave grating and the other eye sees a vertically oriented sine wave grating, people typically see an unstable pattern alternating over time between the horizontal and vertical gratings, interleaved with periods in which a mixed pattern is seen (Figure 2, bottom middle). Finally, in some cases, viewing different luminance/contrast in the two eyes can elicit an enhanced perception of depth, even when binocular disparities are absent [20]. This is referred to as the *sieve effect* (Figure 2, bottom). The perceptual mechanisms that drive the sieve effect are not understood but appear to differ from those that rely on binocular disparities [34]. While luster and binocular rivalry may often be considered undesirable perceptual artifacts, the sieve effect may actually produce a desirable enhancement of 3D percepts.

### 2.2 Dichoptic Tone Mapping Methods

#### 2.2.1 Algorithms.

Several dichoptic tone mapping methods have been proposed in recent years that rely on similar principles [14, 37, 40–42]. Essentially, one eye’s image is tone mapped to maximize the visible contrast across a certain luminance range and the other eye’s image is tone mapped to maximize the visible contrast across a different range.

Yang et al. [37] were the first to propose this method to improve visual richness. They created an optimization algorithm that runs on an image-by-image basis, using an existing standard tone mapping algorithm for one eye’s image and creating a paired image with a different tone map for viewing on a stereoscopic display. The algorithm for generating the second image was designed to maximize the visual differences between the pair of images while maintaining conservative visual comfort and fusion criteria, based on a perceptual model.

This method was followed up on in several additional studies [14, 40, 41]. In particular, Zhang et al. [41] proposed a method for creating dichoptic pairs in which both tone maps can be optimized simultaneously. For this approach, they used an existing bilateral tone mapping algorithm to maximize global contrast in one eye’s image and local contrast in the other eye’s image [12]. The global contrast tone map is obtained by using a filter that maintains sharp edges but smooths out local contrast and therefore substantially lacks detail. This algorithm was used in concert with a perceptual model similar to the one used by Yang et al. [37] to optimize visible contrast and fusibility.

Earlier dichoptic tone mapping methods were not applicable to real-time applications (as is needed for interactive VR experiences), because running the optimization could take several seconds per image [37, 41]. More recent work has thus aimed to speed up the required processing time. Feng et al. [14] proposed a method to ensure temporal continuity for dichoptically tone mapping video frames, but their algorithm still required off-line processing. In a promising development, Zhang et al. [40] recently used GPU acceleration and a neural network approach to substantially reduce processing time, allowing the potential for real-time frame-by-frame deployment. Most recently, Zhong et al. [42] created a dichoptic tone mapping method (referred to as DiCE, for Dichoptic Contrast Enhancement) that uses fixed tone mapping curves for each eye’s image. DiCE tone mapping curves can be applied to either HDR or LDR inputs and are pre-optimized with user studies to reduce binocular rivalry, eliminating the image-by-image optimization step present in previous methods.

#### 2.2.2 User Studies.

The effectiveness of new tone mapping algorithms is often assessed subjectively with user studies in which a group of observers are asked to view a set of example images and either rate or rank them according to some subjective criterion (e.g., References [3, 11, 24, 25, 38, 39]). Two types of procedures are commonly found in subjective image assessments: image ratings (i.e., Likert scales) and forced choice (i.e., comparing images and choosing the best one).

Previous reports evaluating dichoptic tone mapping methods have used two-alternative forced choice paradigms (2AFC) to ask observers to make a choice between a standard tone mapping method (e.g., a non-dichoptic method) and the newly proposed dichoptic method on some image quality criteria. The strength of 2AFC is that it gives reliable and sensitive results [31], but the downside is that it is not always obvious what the best approach is for selecting the *standard* method for comparison.

What is an appropriate standard to compare dichoptic methods against? At the core of dichoptic tone mapping is the notion that the fused image is better than a conventional non-dichoptic image along some desired perceptual dimension, such as image quality or visual contrast. Thus, it is important to establish that a given dichoptic tone mapping method is preferred over a reasonable state-of-the-art non-dichoptic method. However, it is also important to rule out the possibility that either of the component images is consistently preferred over the dichoptic pair. For example, if non-dichoptic viewing of a component image is preferred over viewing the dichoptic pair, it would mean that just one of the tone mapped images (rather than the dichoptic presentation per se) may be driving subjective preferences. However, if dichoptic viewing is consistently preferred over both component images on some desired perceptual dimension, it provides evidence for the theory that binocular fusion can be exploited to increase subjective assessments. That is, that the dichoptic percept benefits from incorporating desirable features from both eyes’ images.

Previous studies have used a range of non-dichoptic standards for comparison during 2AFC tasks, but often omitted direct comparisons with both component images. Yang et al. [37] included a comparison with just one of the component images and found their dichoptic method to be better in terms of visual richness. Zhang et al. [41] used a mixture of standard conditions that included a non-dichoptic tone map (the average of the two component images) and several dichoptic methods. The results suggested that their new dichoptic method was better than the non-dichoptic average in terms of overall contrast, detail, and preference. But no comparisons with the component images were reported. Zhong et al. [42] evaluated both their DiCE method and Zhang et al.’s [41] method against one of Zhang et al.’s component images. With respect to overall image preference, the authors did not find consistent improvement in image quality for either dichoptic method relative to the component. When participants were instructed to make their response on the basis of visual contrast, they found consistent evidence for improved perception of contrast with both methods.

### 2.3 Current Study Motivation

When our own pilot studies began producing negative results for improved image quality and perceived contrast/detail with selected dichoptic methods, we developed a hypothesis that this important choice of the standard for comparison may account for some of these differing results. We thus designed a set of user studies to address this possibility, particularly exploring a range of non-dichoptic alternatives for comparison. We used these studies to evaluate two previously proposed methods: the dichoptic tone mapping methods described in Zhang et al. and Zhong et al. [41, 42], because they provide two recent examples of complementary approaches to tone map generation. In addition, we evaluated a simple exposure-based dichoptic method, as described in the next section.

## 3 EXPOSURE-BASED DICHOPTIC METHOD

To explore additional possibilities for real-time dichoptic contrast enhancement, we tested a method that creates dichoptic images during the capture stage instead of during post-capture tone mapping. This method can serve as a comparison to the more sophisticated tone mapping techniques. Specifically, rather than applying two different tone maps, we simply showed differently exposed images to the two eyes (Figure 1(C)): One eye sees a high exposure image that contains better details in low-light areas, and the other eye sees a low exposure image that contains better details in highlight areas. One benefit of this *dichoptic exposure* method is that it does not rely on having an HDR image as the input and could bypass the HDR imaging and tone mapping pipeline. In this way, it is conceptually similar to computational photography algorithms that generate an HDR image from multiple LDR images with bracketed exposures [6, 29, 30], but we are asking the human visual system to combine two different exposures binocularly, rather than digitally. This process is advantageous for saving computing time during real-time applications (similar to the DiCE method [42]). However, a downside of this exposure-based method is that there is no explicit optimization for reducing perceptual artifacts. Specifically, inter-ocular differences in luminance and contrast are not constrained systematically as in dichoptic tone mapping. Therefore, we would predict that binocular rivalry artifacts might be more likely to occur and reduce perceived image quality. In this sense, this exposure method can serve as a baseline dichoptic method to evaluate the efficacy of the perceptual optimizations performed for other dichoptic methods. In the stimuli for our user studies, we simulated dichoptic exposures, as described in more detail below (Section 4.1.2).

## 4 USER STUDIES

To determine whether dichoptic reproduction methods consistently produce superior images as compared to non-dichoptic methods, we asked participants to subjectively judge the overall image quality of a set of images with different tone maps and exposures applied. We also included judgments based on two more specific criteria: detail visibility and 3D impression. To thoroughly test each dichoptic method, we first conducted a rating study that allowed us to compare dichoptic methods against several non-dichoptic methods in a time-saving manner. The best rated non-dichoptic methods were then directly compared to each dichoptic method in a 2AFC task. Last, to gain a better understanding of the practical implications of dichoptic methods beyond subjective judgments, we conducted an exploratory experiment to assess the visibility of details using an objective performance measure. Overall, we conducted six experiments with a total of 88 participants. The tasks and response criteria used in each of the experiments are outlined in Table 1. All participants had normal or corrected-to-normal visual acuity in both eyes and normal stereo vision as determined by a Titmus test. All participants gave informed consent prior to starting the experiment, were compensated for their time, and were naïve to the study hypotheses and goals. The procedures were approved by the institutional review board at the University of California, Berkeley.

### 4.1 Stimulus Generation

For each experiment, we included several different dichoptic and non-dichoptic conditions (listed in Table 2). By condition, we refer to a method for generating a left/right image pair. Image pairs were generated from natural photographs inline with the stimuli used in previous studies. The image pairs were all generated from a publicly available dataset of HDR images (the HDR+ Burst Photography dataset) [18]. Note that while these image pairs were viewed on a stereoscopic display, because they were generated from a single HDR image they did not have any binocular disparities. This is consistent with the majority of stimuli used in previous dichoptic tone mapping studies [37, 40–42]. In Experiments 1–5, we generated the stimuli for each condition from 18 unique HDR scenes. Because Experiment 6 involved more trials, we used a subset of eight scenes (see Section 4.4.2 for details).

#### 4.1.1 Zhang et al. (2018) Dichoptic Tone Mapping.

**Fig. 3.**
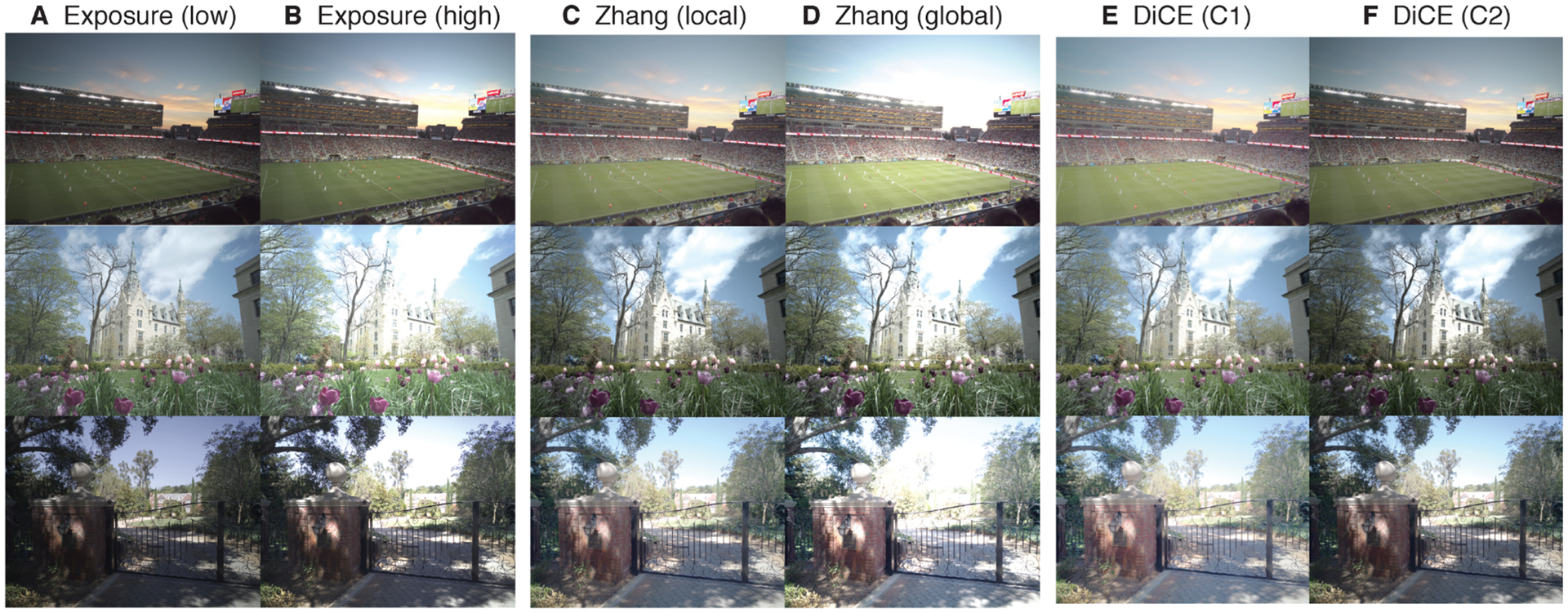
Three example scenes from the HDR+ Burst Photography dataset [18], with each dichoptic image pair illustrated. These include dichoptic exposures (A, B), the dichoptic tone mapping method from Zhang et al. [41] (C, D), and the dichoptic tone mapping method from Zhong et al. (DiCE) [42] (E, F). In A/B, low/high refer to the exposure level. In C/D, local/global refer to the tone mapping algorithm. In E/F, C1/C2 refer to the DiCE tone mapping curves.

Four conditions were selected to examine the perceptual effects of the dichoptic tone mapping algorithm proposed by Zhang et al. in 2018 [41]. The tone mapped images for these conditions were obtained directly from the authors. In the *dichoptic* tone map condition, one eye’s image emphasized local contrast, and the other eye’s image emphasized global contrast (Figures 3(C) and (D)). The details of how these two images were generated is described in the original paper [41]. In brief, the authors optimized a tone mapping parameter that defines the global contrast of a base-layer, which is then combined with the image details [12]. We included three *non-dichoptic* conditions for comparison. In two conditions, both eyes viewed one or the other of the component images that made up the dichoptic pair: We will refer to these two conditions individually as the *local* and *global* conditions. In the *average* condition, both eyes viewed an image generated with the average parameter from the local and global images. This was the standard used for comparison in the original study [41].

#### 4.1.2 Dichoptic Exposure.

We also simulated the proposed dichoptic exposure method and generated a set of non-dichoptic alternatives. One can think of increasing exposure as increasing the number of photons arriving at the camera sensor by some gain factor. To simulate increasing or decreasing camera exposure, we thus applied multiplicative gain factors to the HDR pixel values, with larger gain factors simulating longer exposures. First, we normalized the HDR pixel values to range from 0–1. Next, we applied a gain factor (see below) and gamma corrected by 0.45. We capped any resulting values that were greater than 1 (simulating camera saturation) and converted to 8-bit precision. Following standard practice in photography, we applied a range of gains (from 0.03 to 4, in steps of ~0.2) and selected a proper exposure with minimal clipping (the percentage of pixel values in the lower and upper 4% of the 8-bit range). If multiple exposures of the same scene had similar clipping, we manually selected one. We next simulated a high and low exposure by generating images that were three gain steps above and two gain steps below the proper exposure. These steps were the same for all images and were chosen to maximize differences in detail visibility without a substantial difference in mean luminance. Matlab code for generating this simulation on a typical HDR image is available upon request.

It is important to note that this approach only simulates the proposed dichoptic exposure pipeline. In a real-time application of this pipeline, the two cameras in a stereo camera pair would be simply set to a constant difference in exposure or gain. This could be implemented with a modification to existing automatic exposure methods, whereby after the ideal exposure level is detected, a constant offset is introduced to set one camera slightly lower and one camera slightly higher. The subsequent image capturing would be done without further adjustment or exposure bracketing needed.

Four conditions for the exposure manipulation were generated from these simulations. In the *dichoptic* exposure condition, one eye viewed the low exposure and one eye viewed the high exposure. Example dichoptic pairs are shown in Figures 3(A) and (B). There were three *non-dichoptic* conditions, which were either *proper* exposures, *high* exposures, or *low* exposures.

#### 4.1.3 Zhong et al. 2019 (DiCE).

For Experiments 4 and 5, we also generated conditions to include the recently proposed DiCE dichoptic tone mapping method [42]. We implemented the DiCE algorithm in Matlab and confirmed that the resulting images matched the DiCE test images available online. The original LDR input image for DiCE always had the local tone map from Zhang et al. applied [41]. We used the tone curve parameters from the authors’ main evaluation experiment (their Experiment 2), with a 1/h ratio of 0.63 and two segments for each tone curve. We included the full *DiCE* dichoptic pair, as well as each component tone map, which we refer to as *C1* (better contrast for low-lights) and *C2* (better contrast for highlights). Example dichoptic pairs are shown in Figures 3(E) and (F).

#### 4.1.4 Stimulus Presentation.

For all experiments, a mirror haploscope with two LCD displays was used to present separate images to the left and right eyes. The displays (DELL U2415) had a pixel resolution of 1920 × 1200, screen size of 52 cm × 32.5 cm, and refresh rate of 60 Hz. The maximum luminance was approximately 174 cd/m^2^. During the experiments, the participant sat in a dark room and viewed the displays from approximately 60 cm away with their head on a chin rest. The experiments were controlled by Psychtoolbox [23] in Matlab and responses were made on a keyboard.

### 4.2 Experiments 1, 2, and 3: Preference, Subjective Detail Visibility, and 3D Impression Ratings

In the first three experiments, we re-evaluated the dichoptic tone mapping method proposed by Zhang et al. [41] along with the new dichoptic exposure method using a set of image rating tasks. Instead of doing a 2AFC task with a single non-dichoptic standard, we asked participants to rate the images one at a time, allowing us to efficiently compare dichoptic methods with multiple non-dichoptic conditions [31].

#### 4.2.1 Participants.

Sixteen adults participated in both Experiments 1 and 2 (13 F, 3 M, age range 19–30). The order of the experiments was randomized such that half of the participants completed Experiment 1 first and the rest completed Experiment 2 first, with at least one week in between. Sixteen additional adults participated in Experiment 3 (13 F, 3 M, age range 18–40).

#### 4.2.2 Procedures.

In Experiment 1, participants were instructed to rate a series of images based on their overall preference on a scale of 1 to 5, with 5 being best. The specific instructions were to rate based on their “preference for image quality captured by the camera and not the scene content.” Prior to starting the experiment, participants were briefly shown each stimulus once to give them an understanding of the range of quality. There were 144 unique stimuli (8 conditions × 18 scenes) presented in randomized order, and each stimulus was shown twice for a total of 288 trials. During the repeated trials, we switched which eye saw which image. For example, for the *dichoptic exposure* condition, on half of the trials the left eye saw the high exposure and on the other half of the trials the right eye saw the high exposure. The presentation time for each stimulus was 6 seconds. In the Supplementary Material, we provide an analysis of potential contributions of left or right eye dominance to user preferences. Consistent with prior work, we do not find evidence for a substantial contribution of eye dominance, so we averaged across the two repeats for the main results [37, 42].

The procedures for Experiments 2 and 3 were the same as Experiment 1, except that the instructions differed. In Experiment 2, participants were instructed to rate based on their “impression of detail visibility across the scene.” During the instructions, they were shown an example scene (not from the actual experiment) to explain the concept of detail visibility. We showed them a low exposure, high exposure, and proper exposure of this scene, pointing out the details in the highlights and low-lights. We refined this method over pilot testing to ensure that the naïve participants understood the concept of image contrast and visible detail. In Experiment 3, participants were instructed to rate how 3D each scene looked. They were told that “we are interested in whether certain types of image adjustments look more 3D to you. Some images might have worse quality in terms of visible details, but please only focus on how 3D each scene appears.” We refer to participants’ responses to this prompt as their *3D impression*. While 3D enhancement is not the stated goal of dichoptic tone mapping and exposure, we included this task because previous work, and our own pilot testing, suggested that these methods can create an enhanced 3D impression (perhaps related to the sieve effect) [42].

#### 4.2.3 Results for Dichoptic versus Non-dichoptic.

We analyzed the participant ratings first with a Friedman test to separately compare the tone mapping conditions and the exposure conditions. The results showed that there were significant differences among the groups of conditions across all three experiments (Table 3). We also found that there was overall high agreement across participants (as determined by Kendall’s coefficient of concordance, Table 3), except that there was only moderate agreement for the ratings of overall preference in the exposure conditions. In this main analysis, we focus on the raw ratings, however, we include an analysis of differential mean opinion scores (DMOS) in the Supplementary Material.

**Fig. 4.**
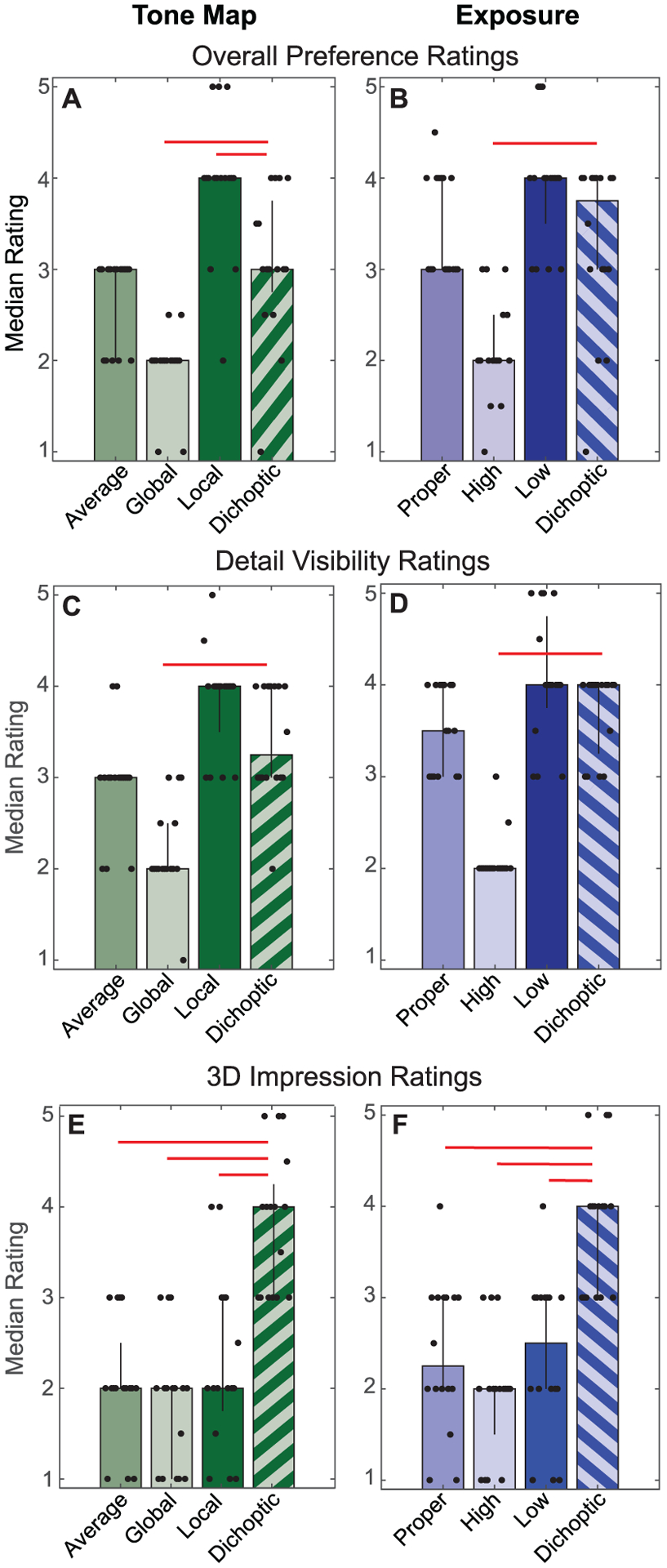
Results of rating tasks in Experiments 1, 2, and 3. The top panels (A, B) correspond to the overall preference ratings for the tone mapping (A) and the exposure (B) manipulations in Experiment 1. The middle panels (C, D) correspond to the perceived detail ratings in Experiment 2, and the bottom panels (E, F) correspond to the 3D impression ratings in Experiment 3. Each black dot represents an individual participant’s median rating for that task and condition (labeled on the x-axis). Dots are jittered for visibility. Each bar represents the median rating across all participants’ medians. Error bars indicate the 75th and 25th percentiles. Red lines mark statistically significant pairwise comparisons (see Table 4).

The results for overall preference (Experiment 1) and detail visibility (Experiment 2) are plotted in Figures 4(A)–(D) and pairwise statistical tests are reported in Table 4. Starting with the four tone mapping conditions (Figures 4(A) and (C)), we did not find evidence that images in the dichoptic condition were consistently rated higher than the highest rated non-dichoptic condition. When compared to the two component images, the dichoptic condition was rated significantly better than the global tone map in both Experiments 1 and 2, but was rated lower than the local tone map (this difference was only statistically significant in Experiment 1). The low ratings for the global tone maps are not too surprising. Viewing these images with smoothed local contrast likely does not result in good subjective image quality unless they are combined with more local detail. It is perhaps also not surprising that the local tone map exceeded all other conditions for the detail visibility rating, since this tone map prioritizes local detail. Ideally, however, a dichoptic tone map would completely, or nearly completely, preserve the visibility of local details. Some reduction in detail might be desirable so long as overall image quality was improved with the dichoptic tone map; however, we also found that the local tone map exceeded all other conditions in terms of overall preference (Figure 4(A)). This result suggests that the inclusion of the global tone map in the dichoptic condition did not increase users’ overall preferences. Ratings of dichoptically tone mapped images were not significantly different than the ratings for the average tone map.

Next, we turn to the exposure conditions (Figures 4(B) and (D)), for which we again did not find a consistent dichoptic preference. The median rating for the dichoptic exposure was descriptively higher than the median rating for the proper exposure, however this difference was small and not statistically significant. As compared to the two component images, the dichoptic exposure was rated significantly higher than the high exposure, but did not differ significantly from the low exposure. These results suggest an overall preference of the participants for underexposed images as compared to overexposed images, perhaps because the underexposed images offered a more appealing balance of light and dark points [16]. Again, we found that the ratings of the dichoptic condition did not systematically exceed the ratings for the most preferred non-dichoptic component image (in this case, the low exposure).

The results from the 3D impression ratings in Experiment 3 differed notably from the other two experiments (Figures 4(E) and (F)). These results supported the observation that dichoptically tone mapped/exposed images create an enhanced 3D impression. For both the tone mapping and exposure conditions, participants consistently rated the dichoptic conditions to be more 3D than each of the non-dichoptic conditions (these comparisons were all statistically significant). The 3D impression results stand in contrast to the results from the other two experiments: They suggest that dichoptic methods can generate a substantially different perceptual experience than non-dichoptic methods. However, participants seem to attribute this experience to a difference in 3D information rather than an increase in visible details.

#### 4.2.4 Results Across All Conditions.

Recall that our baseline dichoptic exposure method did not incorporate strategies to reduce perceptual artifacts, such as binocular rivalry. Thus, it is also interesting to examine how well this exposure method compares to tone mapping methods optimized to reduce artifacts. In these experiments, the dichoptic exposure method was not rated significantly different for overall preference (*z* = −1.46, *p* = 0.14) or 3D impression (*z* = 0, *p* = 1) as compared to the optimized dichoptic tone mapping method from Zhang et al. [41]. The exposure method was rated significantly higher for detail visibility (*z* = −2.27, *p* = 0.02). These results suggest that a relatively naïve dichoptic method may be sufficient to obtain the gains in 3D impression, although it is key to consider how important these gains are for stereoscopic content (see Discussion).

Last, to ask whether there is a best tone mapping method based on these results combined, we also descriptively compared the rankings among all eight conditions. For overall preference, the highest rated method was tied between the local tone map and the low exposure. For detail visibility, the highest rated method was tied between the local tone map, the low exposure, and the dichoptic exposure. For 3D impression, the highest rated method was tied between the dichoptic tone map and dichoptic exposure. These rankings highlight the fact that different methods may be more suitable for supporting different aspects of perceptual experience. While overall preference may be the best global criterion to use for many applications, it is interesting to consider whether it is useful to combine multiple criteria when picking a tone mapping method. As a first step towards a multi-dimensional approach, we also calculated an overall rating score for each method by simply summing the median ratings across the three experiments. Considering all three criteria in this way, the best method would be dichoptic exposure, with an overall median score of 11.75 out of 15 possible. Importantly, this approach gives equal weight to all three criteria, which may not be appropriate. However, in some cases it might be possible to select a set of criteria and weights for a specific application. For example, if a compromise on detail visibility were desirable to obtain an enhanced 3D impression, both dichoptic methods would likely be more desirable than any non-dichoptic ones.

#### 4.2.5 Results Summary.

With respect to overall preference and detail visibility, our results suggest that both dichoptic methods were better on average than one of their non-dichoptic component images (global tone map and high exposure), but not the other (local tone map and low exposure). These results suggest that dichoptic methods may not yet consistently yield improvements along these perceptual dimensions, which differs from the conclusions drawn from previous user studies [37, 40–42]. However, when 3D impression is considered as well, we found more evidence to support substantial subjective improvements with dichoptic tone mapping. The difference in our conclusions with respect to prior work might be explained by the decreased sensitivity of the rating task as compared to the 2AFC tasks used in previous studies. However, the current results also suggest another potential explanation: that the preferences for the dichoptic methods over some standards may be explained by people preferring one component image of the dichoptic pair, rather than the dichoptic pair *per se*.

### 4.3 Experiments 4 and 5: Preference and Subjective Detail Visibility Forced Choice Comparisons

For the next set of experiments, we focused in on comparing the higher rated non-dichoptic conditions against the dichoptic conditions with a 2AFC task. This allowed us to test the hypothesis generated by the previous experiments with a targeted comparison. We only focus on preference and detail visibility, as the results for 3D impression were quite clear. For these experiments, we created four dichoptic versus non-dichoptic 2AFC comparisons. These included the dichoptic conditions from the previous experiments and one new one. While earlier experiments were in progress, a new dichoptic tone mapping method was published by Zhong et al. [42], called DiCE, which we incorporated into these next studies.

#### 4.3.1 Participants.

Two different groups of 16 adults participated in these studies. One group completed the preference task (10 F, 6 M, ages 18–23) and the other group completed the detail visibility task (11 F, 5 M, ages 18–28).

#### 4.3.2 Procedure.

The paired conditions for comparison are shown in Table 5. For the Zhang et al. [41] dichoptic tone map and for the dichoptic exposure conditions, the non-dichoptic standards were selected as the most preferred component images from Experiments 1 and 2. For the DiCE conditions, we included comparisons with both component images, because we did not have ratings data to justify choosing one over the other.

On each trial, participants viewed a dichoptic and a non-dichoptic pair of the same scene in sequence, for 3 seconds each. They indicated whether they preferred the first or the second image in terms of overall image quality (Experiment 4) or detail visibility (Experiment 5), using the same basic instructions from Experiments 1 and 2. There were four repeats of each comparison, for a total of 288 trials. Before starting the experiments, participants viewed all stimuli once.

#### 4.3.3 Results.

**Fig. 5.**
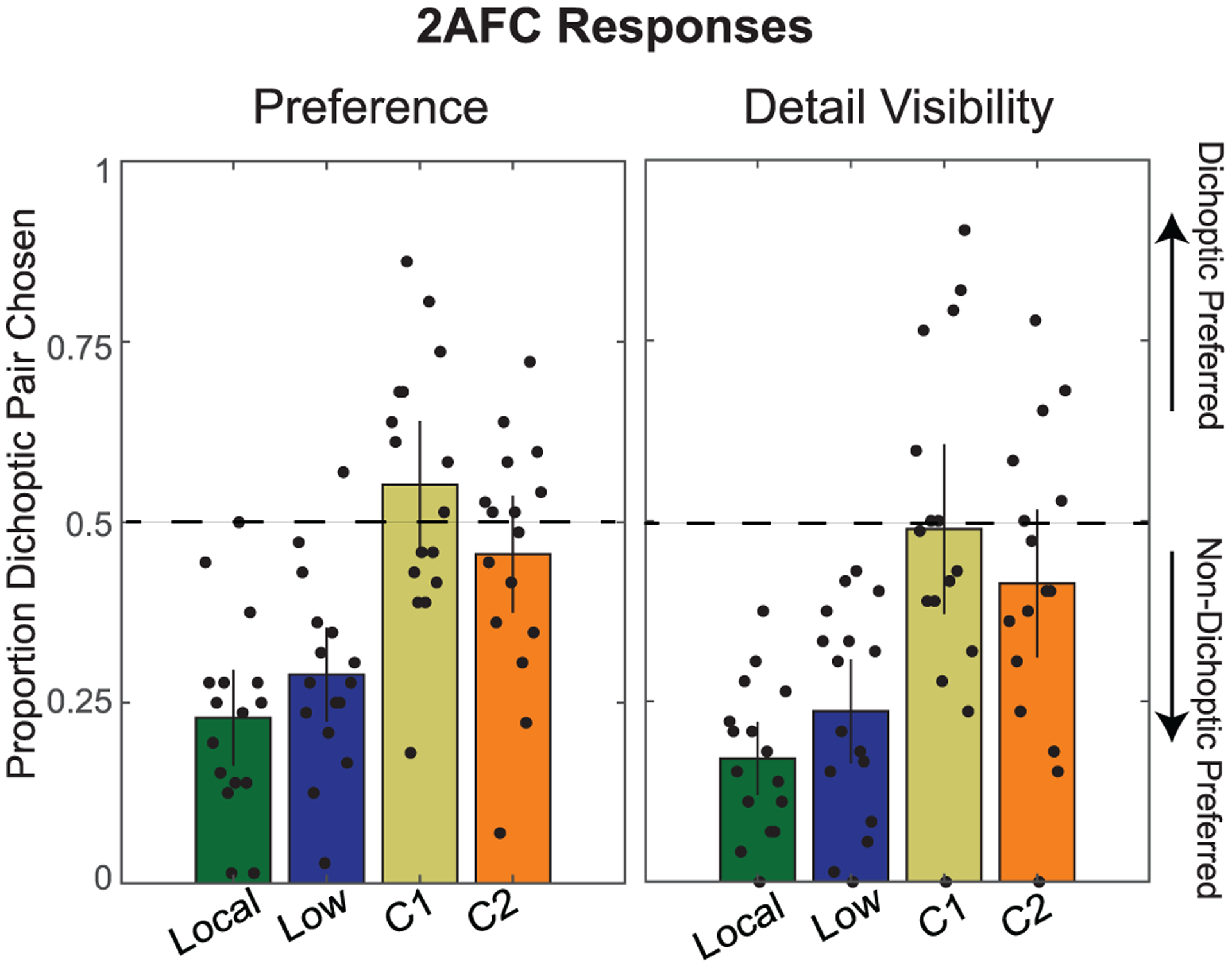
Results from 2AFC task in Experiments 4 (left) and 5 (right). Each black dot represents an individual participant’s responses, in terms of proportion of trials that the dichoptic condition was chosen over the non-dichoptic component image (higher indicates that dichoptic is better). Dot locations are jittered for visibility. Each bar represents the overall mean proportion across all participants. Error bars indicate 95% confidence intervals. The black dotted horizontal line indicates chance level.

For both experiments, we calculated the mean proportion of trials in which each participant chose dichoptic over non-dichoptic images. The results are plotted in Figure 5 and statistical tests are summarized in Table 6. In the figure, a preference for the dichoptic method is indicated by data points falling above the dashed line. Qualitatively, in both experiments there was no consistent preference for dichoptic viewing compared to non-dichoptic viewing of the component images across all four conditions. The only consistent preferences were in favor of the non-dichoptic component images. The results from the Zhang et al. [41] tone map and the exposure conditions support the rating results from Experiments 1 and 2, which suggested that participants prefer the better quality component image of the dichoptic pair over dichoptic viewing. The dichoptic pair in these comparisons was selected at a rate significantly lower than chance in both Experiments 4 and 5. Once again, this is perhaps not surprising for the detail visibility judgment on the images from Zhang et al., however, the fact that the same results were obtained for the overall preference judgment suggests that more work is needed to ensure that dichoptic tone maps boost subjective image quality beyond the components.

The results for the DiCE method are closer to chance on average and highly variable across participants. Statistical tests suggested that DiCE dichoptic images were chosen at levels that did not differ significantly from chance in both experiments. While a few participants had a consistent preference for DiCE over one or the other component, these effects were counterbalanced by other observers having a consistent preference against DiCE.

In summary, the results of these 2AFC tasks (in concert with the ratings experiments) suggest that the study participants did not have a strong, consistent preference for dichoptically tone mapped images with respect to overall image quality or detail visibility as compared to the images tone mapped with conventional non-dichoptic methods.

#### 4.3.4 Scene-based Analysis.

**Fig. 6.**
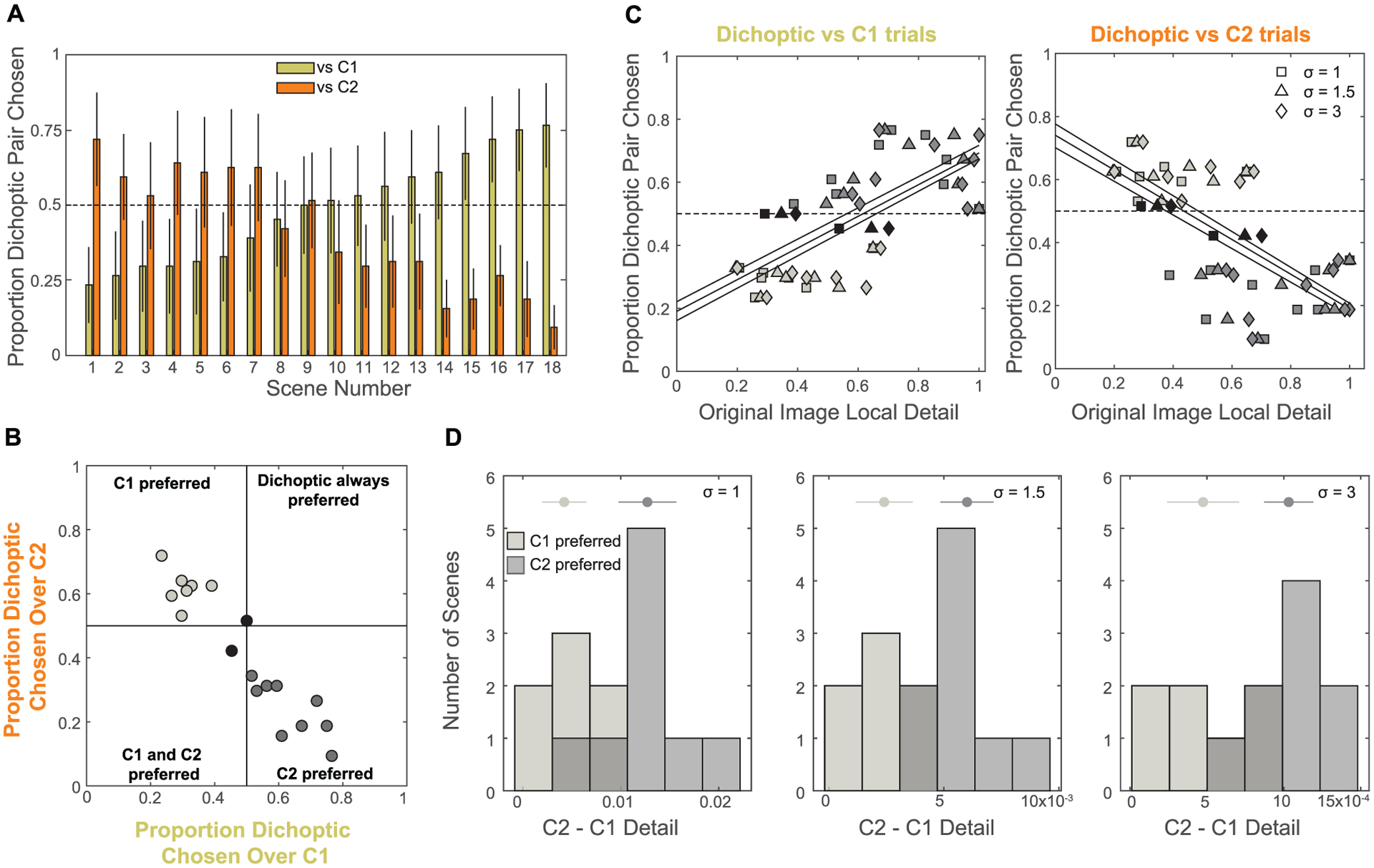
Results of exploratory scene-based analysis on Experiment 5. (A) We re-analyzed the results for the 2AFC local detail task on the DiCE-generated images separately for each scene. Each bar indicates the proportion of trials for which the dichoptic pair was chosen versus each component (C1, C2), averaged across participants. Error bars show the 95% confidence intervals. Data are sorted by the C1 comparisons. (B) Each point represents the dichoptic preference of an individual scene when compared to C1 (x-axis) and C2 (y-axis). (C) To quantify local detail (x-axis), we converted each image to grayscale and convolved with a rotationally symmetric Laplacian of Gaussian filter. We took the mean of the absolute value of the filter response and normalized by the mean pixel value to account for decreased perceptual sensitivity to contrast at higher luminance levels. We repeated this analysis with filter standard deviations (*σ*) of 1, 1.5, and 3 pixels, represented by different symbols. Black lines show best-fit linear regressions for each filter size. The response range of each filter size was normalized to range from 0 to 1 to allow plotting on the same axes. (D) For each filter size, each plot shows a histogram of the difference between the C2 and C1 image local detail, separated out by C1-preferred and C2-preferred images. Above the bars, the mean and 95% confidence intervals are shown for the C1-preferred and C2-preferred scenes.

We were curious if different scenes might lend themselves more or less to dichoptic methods. For this exploratory analysis, we focused on the results obtained from the DiCE method, because this approach produced a mixture of preferences that were greater than and less than chance across different participants (Figure 5). We also focused on the detail visibility judgment, because this perceptual task is most closely related to contrast enhancement. When we analyzed the data separately for each scene, we found that different scenes produced consistently different preferences in the 2AFC task. Figure 6(A) shows the proportion of trials on which the dichoptic pair was chosen for each scene when compared to the components (C1 and C2), averaged over all participants. Across different scenes, these averages varied from lower than chance to greater than chance. As shown in Figure 6(B), we observed a negative correlation between the responses when dichoptic pairs were compared to C1 and C2 (r = −0.94). When the dichoptic pair was consistently chosen more than C1, it tended to be consistently chosen less than C2, and vice versa (upper left and lower right quadrants). No scenes were consistently preferred with the dichoptic method when compared to both components.

We next asked whether this pattern could be explained by any low-level features of the images. We found that the participant preferences were strongly correlated with the amount of local detail in the original images (Figure 6(C), original image before applying DiCE tone maps). Local detail was quantified as the mean absolute response of a Laplacian of Gaussian filter, and this relationship was consistent over a range of filter sizes (see figure caption for details and Table 7 for statistical analysis). Higher levels of local detail in the original image were associated with a tendency for participants to prefer the dichoptic pair versus C1 (left), but also with a tendency for participants to prefer the C2 over the dichoptic pair (right). We categorized the scenes as either C1-preferred (light gray markers/bars) or C2-preferred (dark gray markers/bars). Two scenes without consistent preferences were excluded from this analysis (black markers). For each scene category, we calculated the difference in the local detail of the C2 and C1 images. We found that C2-preferred scenes tended to have higher detail in the C2 images than C1-preferred images; C1-preferred scenes tended to have similar levels of detail between C1 and C2 (Figure 6(D), Table 7). These results suggest that low-level image properties can reliably predict which non-dichoptic component image would be preferred over the dichoptic pair, but they do not yet tell us why (see Discussion). This is the first analysis that we are aware of that examines image-based predictors for dichoptic tone mapping preferences based on user study data. Moving forward, image-based analyses of larger datasets that cover a diverse range of scenes may be able to help guide further algorithmic development for dichoptic tone mapping.

### 4.4 Experiment 6: Objective Detail Visibility

The potential use cases of VR are varied and extend beyond situations in which simply enhancing subjective image quality is the goal. For example, if dichoptic methods can objectively increase visible detail in a scene (without subjective improvement), then they may be quite useful for applications such as remote guidance.

Objective perception of visibility in natural images is challenging to predict, and there exists no standard technique for characterizing how detail visibility varies across a natural image. For example, it was recently shown that detection of small targets embedded in natural images depends on several scene properties, such as luminance, contrast, and pattern similarity [33]. We thus designed a novel exploratory task that we predicted would be easier to perform if details were more visible in natural images. Specifically, we showed people images and cued them to look at a specific region. We then presented them with two probes, which were taken from a small patch in that region. One probe was consistent with the original scene, and the other probe was mirror-reversed about a vertical axis. We asked participants to indicate which one matched the original scene. Our reasoning was that if a particular condition resulted in better perceived visual detail, then participants should be able to better discriminate the original patch from a mirrored one. Note that this reasoning is only valid if the patches have at least some visual detail within them, and if they are not perfectly left/right mirror symmetric.

#### 4.4.1 Participants.

Twenty-four adults participated (19 F, 5 M, ages 18–34).

#### 4.4.2 Stimuli.

**Fig. 7.**
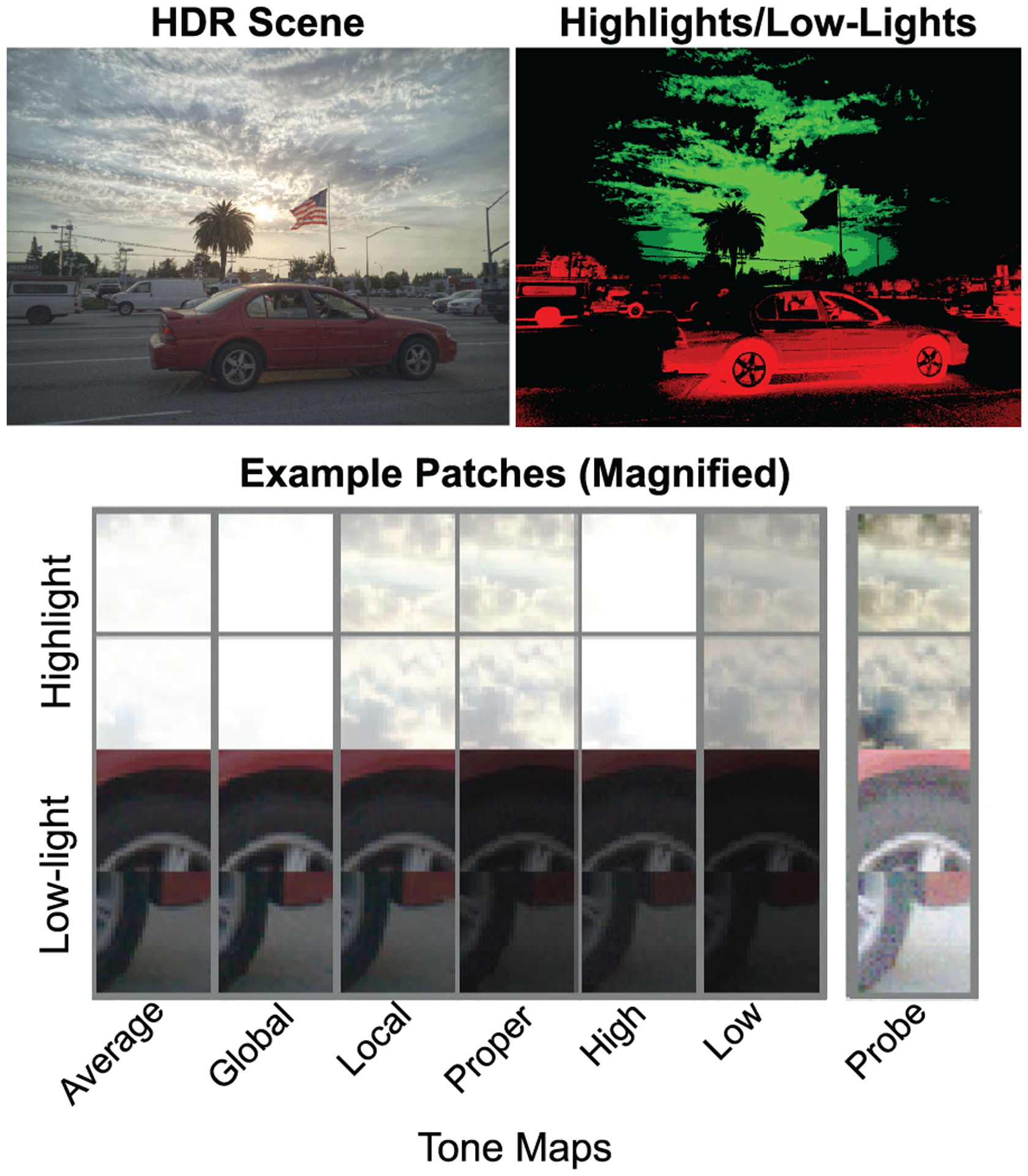
Example of an HDR image (from the HDR+ Burst Photography Dataset [18]) and its intensity map, containing highlight and low-light areas. Highlight areas are color coded in green (e.g., sky) and low-light are coded in red (e.g., the car). Color saturation is used to illustrate the strength of the high/low-light. Examples of test patches for two highlight and two low-light areas are shown below. The first six columns show how the patches are rendered in the original scene under different conditions. The last column shows the probe patch used, for which a local tone map has been applied.

In this experiment, we used the same eight tone mapping and exposure conditions as in Experiments 1–3. Usually highlight and low-light areas are the most difficult to reproduce on a conventional display, so we focused on these areas for our stimuli. We defined highlights and low-lights broadly as the top and bottom 15% of pixel values according to their intensity in grayscale in the original HDR images (illustrated in Figure 7). We then segmented these images into 35 by 35 pixel patches and selected eight highlight-dominant and eight low-light-dominant patches from each scene. Highlight and low-light patches were defined as patches in which at least 75% of the content was made up by high- or low-light pixels. In cases where a scene contained more than 16 patches that met these criteria, we manually chose a subset with minimal mirror symmetry. The bottom panel in Figure 7 shows examples of what these patches looked like when each of the tone maps used in this experiment were applied to the HDR image.

For each patch, we next created a *probe* version to test performance on the orientation task. This probe was created with a custom, purely localized tone map applied to the HDR values within the specific intensity range of each patch, using Matlab’s *imadjust* function (Figure 7, right column). This approach provided a way to test performance with the same probe that differed from all the specific tone maps being assessed. Visualizations of all patches and probes are available upon request.

##### Procedure.

**Fig. 8.**
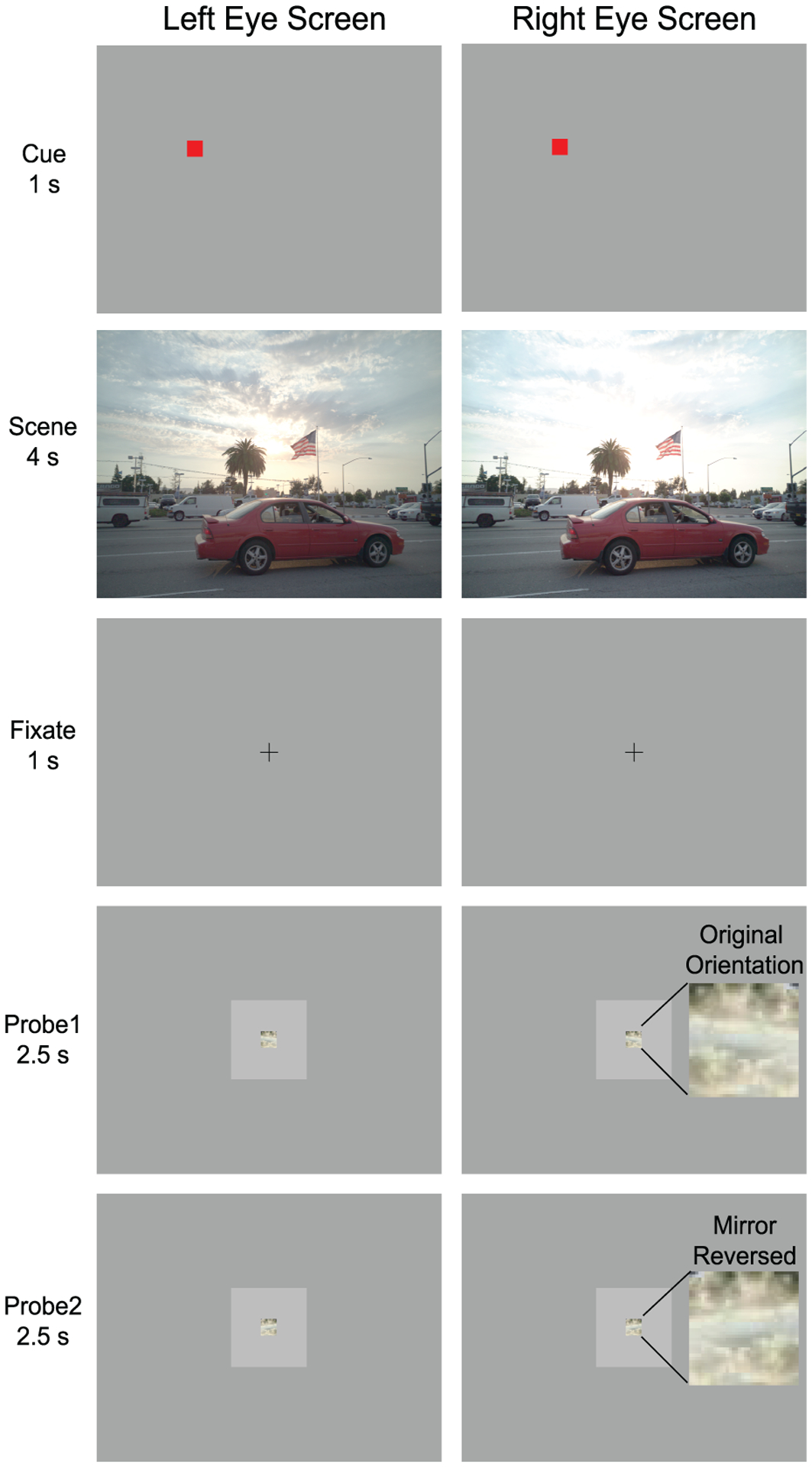
A schematic of a trial in the objective detail visibility task in Experiment 6. Magnified views of each probe are shown on the right in the lower two rows. In this example, Probe 1 matches the orientation in the original scene and Probe 2 is mirrored.

The procedure for one trial is illustrated in Figure 8. The participant was first presented with a red box cuing a target area on the screen (~1 deg visual angle) for 1 second. Then the cue disappeared and an image (dichoptic or non-dichoptic) from a particular scene was shown for 4 seconds. The participant was asked to look at the cued location in the scene and study that area very well. Then the scene disappeared and a fixation cross was shown for 1 second at the center of the screen. After the fixation cross disappeared, two probe patches were shown consecutively for 2.5 seconds each. One probe was always in the original orientation and one probe was mirror reversed, in random order. The participants were told that the probe patches could have different contrast, brightness, and color compared to what they saw in the original full scene, so they were instructed to focus on visual patterns within the location. The patches were shown on a mean luminance background against a screen of mid-gray (83 cd/m^2^). Due to a slight offset in the alignment of some images obtained from Zhang et al. [41], which was discovered after completing data collection, the cued location was slightly lower than the selected patch in some of these conditions. This offset was small (about 5 pixels) and is not expected to influence the results.

Following a Latin Square design, each participant saw a predetermined set of scene/condition combinations, where each scene was only viewed in one condition. This design prevents participants from remembering the scene content that may be visible in one condition but not another, while still randomizing the assignment of scenes to conditions across all participants. We used the same eight conditions as in Experiments 1–3. Specifically, two dichoptic methods (Zhang et al. dichoptic tone mapping [41] and dichoptic exposure) were included, along with non-dichoptic versions of the component images, and two additional non-dichoptic standards (average tone map and proper exposure). Each particular scene/condition combination was seen by three different participants. For example, Participants 1, 9, and 17 all saw Scene 1 in the average tone map condition only, and Scene 2 in the global tone map condition only, and so on. For each scene, there were eight low-light and eight highlight trials, creating a total of 128 trials.

#### 4.4.3 Results.

**Fig. 9.**
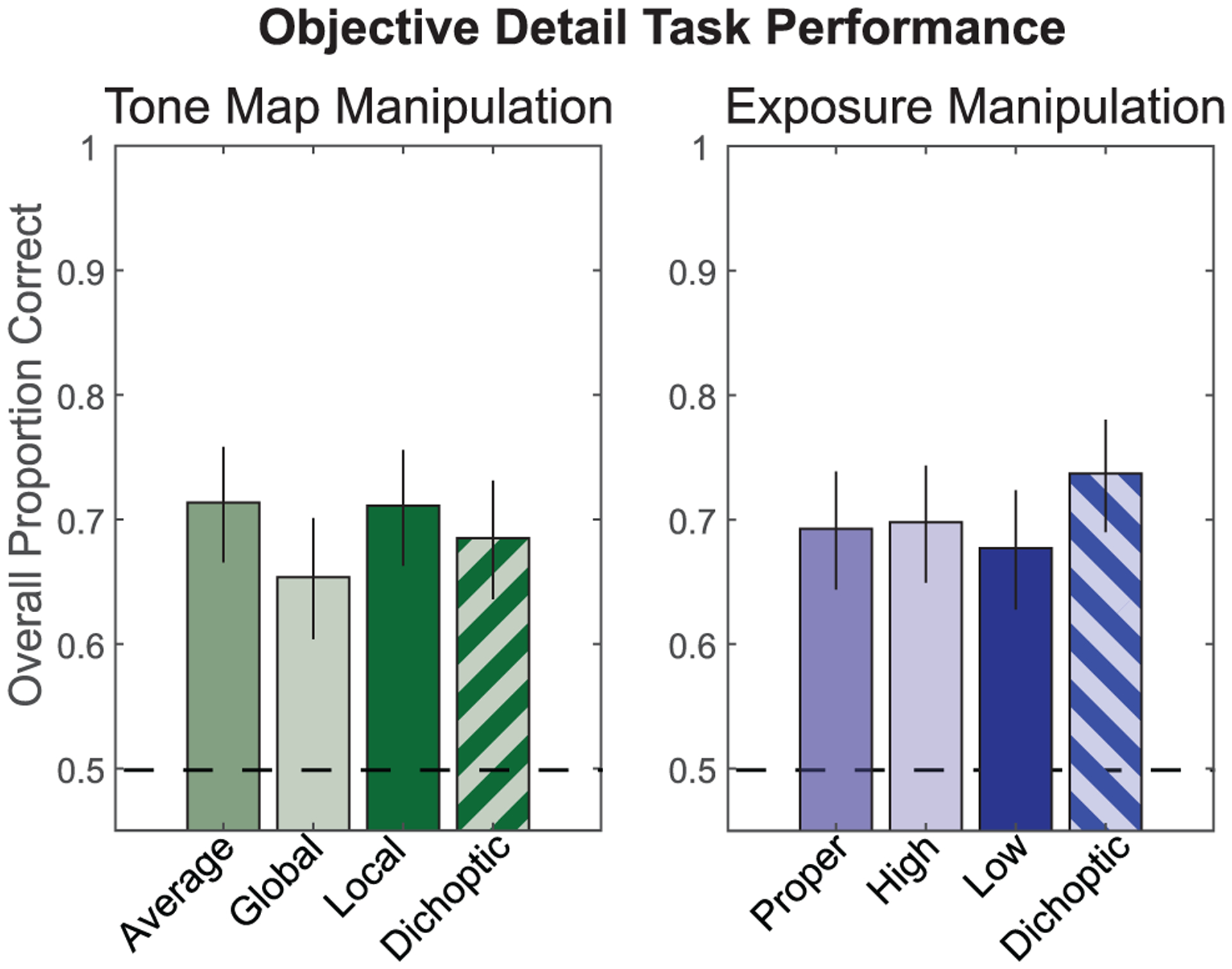
Results of the objective detail visibility task in Experiment 6. Each bar represents the proportion correct across all participants for each condition (labeled on the abscissa). Individual data are omitted, because each participant only saw a subset of scenes in each condition. Error bars indicate 95% confidence intervals calculated from the binomial distribution. The dashed line indicates chance performance.

Figure 9 shows the performance on the orientation discrimination task calculated over all participants in terms of the proportion of correct responses. Performance was above chance in all conditions, suggesting that the task was challenging, but not impossible. Qualitatively, we did not observe any substantial differences across conditions.

To examine potential statistical differences between conditions, we fit the trial-by-trial data using a logistic regression model. First, we focus on the results for the tone mapping conditions (Figure 9, left panel). We modeled the four viewing conditions as categorical predictors and modeled both participant and scene as random effects. As in the analysis of the subjective data, we focus our analysis on differences between the dichoptic condition and the other three conditions. In the upper panel of Table 8, we report the coefficient estimates, odds ratios, and p-values associated with the model. Odds ratios were determined by exponentiating the coefficient estimates, and can be interpreted as the increase or decrease in the probability of a correct response associated with these non-dichoptic conditions, as compared to the dichoptic condition. Values less than 1 indicate that a given condition resulted in fewer correct responses than the dichoptic condition, and vice versa. The p-values show that there were no statistically significant differences with the dichoptic condition. However, the performance when viewing the global tone map approaches being significantly worse than performance when viewing the dichoptic tone map. This makes sense, given that the global tone mapping algorithm is optimized for preserving overall global, rather than local, contrast [12].

The results for the different exposure conditions (Figure 9, right panel) were analyzed in the same manner (Table 8, lower panel). We did not observe any statistically significant differences in performance between the dichoptic exposure condition and any of the non-dichoptic conditions.

The premise for this experiment is that the performance on the patch orientation judgment task should be modulated by perceived contrast. Specifically, higher perceived contrast should lead to better performance, because observers are better able to distinguish between the original and mirrored patches. In the limit, the task should be impossible if a patch is perceptually uniform. However, it is important to evaluate this premise on the specific stimulus set used. With this goal, we ran a post hoc analysis on the data. The details of the analysis are reported in the Supplementary Materials. The results suggest that, while contrast was correlated with performance on this task, the relationship was highly nonlinear. Specifically, higher contrast was associated with better performance for low contrast patches, but above a certain threshold this relationship was no longer reliable. At these supra-threshold levels, other factors of the stimulus, such as the horizontal mirror symmetry or the distinctiveness from the surrounding area, likely contribute to variability in performance. A definitive answer to the question of whether dichoptic tone mapping increases objective detail visibility will likely require a more challenging task that is consistently modulated by stimulus contrast across a broad range.

## 5 DISCUSSION

### 5.1 Relationship to Prior Work

The current experiments did not include all published dichoptic methods (e.g., References [14, 37, 40]) and should be interpreted in combination with the results from other studies [37, 40–42]. The efficacy of a dichoptic method is always judged relative to the standard used for comparison. Our study used non-dichoptic standards that are different from most of the prior work, since we were interested in how well dichoptic methods perform compared to their component images. The most similar data from prior work were obtained in the study by Zhong et al., in which the authors assessed Zhang et al.’s dichoptic method against the local tone map [42]. Our result is consistent with their finding that the dichoptic tone map was not chosen over the local tone map for overall preference. However, contrary to their findings, our data also suggest that the dichoptic tone map is not consistently preferred when judging detail visibility (in their user study, the dichoptic tone map was preferred over the local tone map in terms of contrast). While related, prompts to judge based on detail or contrast may be interpreted differently by participants, which may contribute to these different results. The other work that compared a dichoptic tone map against one component image is by Yang et al., but we did not include their tone mapping approach in our studies [37]. They found that the dichoptic method was consistently preferred based on “visual richness and content clarity.” Based on the pattern of results in our study, it is possible that subjective assessments of visual richness may incorporate effects of dichoptic tone mapping on 3D impression in addition to perceived contrast and detail.

In addition to different non-dichoptic standards and response prompts, there is a range of other experimental factors such as image sets and stimulus presentation time that differ across published studies and could affect the judgments of participants. In practice, the choice to adopt a dichoptic tone mapping algorithm may depend on balancing the range of desired perceptual effects and the tolerance for introducing artifacts such as rivalry. Taken together, these results emphasize that the choice of the baseline method for comparison and the perceptual criterion may influence the conclusions drawn from user studies. An assessment including both component images can provide a more complete picture for judging a dichoptic methods’ performance. For example, the results from Experiment 3 show a compelling case in which the percept of the dichoptic pair exceeds the quality of both components individually.

### 5.2 Binocular Contrast Perception in Natural Images

Given the psychophysical work on binocular contrast combination, why might our study suggest that dichoptic tone mapping methods perform inconsistently? Psychophysical experiments on binocular contrast combination, which support a nearly winner-take-all model, tend to use simplified visual stimuli, such as sine wave gratings [26]. In natural imagery, it is possible that interactions between different spatial frequencies and other contextual information have an effect on binocular perceived contrast. For example, a recent study reported that binocular contrast perception can be dominated by the eye that sees the lower contrast stimulus (loser-take-all) when accompanied by local boundary information similar to what might occur at object boundaries in natural images [17]. This result suggests that context can strongly modulate binocular contrast perception.

Images of natural scenes also vary in terms of their local spatiotemporal properties. It is thus important to consider whether user study results could be specific to the selected images. For example, scenes that lack a high dynamic range may not require more than one tone map to capture visible contrast. While we do not have an absolute measure of luminance levels in the dataset that we used [18], the scenes were chosen to be diverse in content and dynamic range, with outdoor scenes that include both sky and shadows [36]. A follow-up analysis of the variability of responses across scenes suggests that the overall conclusions of Experiments 4 and 5 are consistent even with smaller image samples (see Supplementary Material). Furthermore, our analysis based on image-computed local detail suggests that a large amount of variability across images is predictable: Much of the variability in our data is accounted for by the local detail of the original image. The two DiCE tone mapping curves differentially allocate emphasis on local detail across different portions of the image histogram (low-lights or highlights). Thus, the current results support the idea that simple image-computable measures of visual contrast can be predictive of perceptual judgments of natural images. However, the image measures that need to be optimized for dichoptic imagery are still not well understood.

Moving forward, it would be interesting to examine the rules of contrast combination in stimuli of intermediate complexity (for example, small patches of natural images), in which the effects of image content and context can be studied in more detail (for example, see Reference [9]). A better understanding of how image features relate to perceived contrast might yield insights for using these features to effectively modulate a dichoptic tone mapping operator. For example, people may select particular salient features in a scene to make their decision about image quality. People may also tend to place different levels of emphasis on details in low-lights or highlights. Details in the shadows may be considered less important for taking in the content of a scene. Studying dichoptic tone mapping of smaller patches of natural images may help clarify some of these issues by reducing the number of different features in each unique stimulus.

### 5.3 3D Imagery

The stimuli used in our experiments were not stereoscopic. In this regard, our stimuli were not representative of typical imagery viewed on VR and other stereoscopic displays, in which binocular disparities are leveraged to create a richer 3D percept. This choice was made to match the methods of prior work as closely as possible, but it also poses a potentially important limitation for inferring the effects of dichoptic tone mapping in VR. We can, however, draw some inferences from prior work about how binocular disparities might interact with dichoptic tone mapping in natural imagery. To our knowledge, the only dichoptic tone mapping study that included stereoscopic imagery was the study by Zhong et al. [42]. Their assessment of perceived contrast and 3D impressions in stereoscopic VR was consistent with their findings with non-stereo content. From the vision science literature, simple gratings have been used to study how binocular offsets (i.e., phase shifts between gratings shown to the two eyes) affect binocular contrast perception [8]. According to this work, adding a phase shift between gratings presented to the two eyes does not substantially change binocular contrast perception unless stimuli have low contrast and short presentation times (around 100 ms).

Based on these previous studies, it seems unlikely that the current results of our study for detail visibility would be qualitatively different if stereoscopic images were used. However, this assumption should be tested. Interestingly, binocular contrast differences between gratings do have a robust effect on binocular phase perception (i.e., the perceived phase of the binocularly viewing sine wave stimulus) [10]. This finding suggests that dichoptic tone mapping could have complex effects on perceived 3D shape, location, and layout. A characterization of how dichoptic contrast differences interact with stereo depth in natural images is a valuable topic for future research.

### 5.4 Objective Visual Performance-based Metric

Last, to probe the impact of dichoptic methods on perceived detail, we introduced an exploratory performance-based task. However, this task did not reveal substantial differences in perceived detail between the dichoptic conditions and the non-dichoptic conditions. It is important to consider that this negative result could be due to the task itself. While we were attempting to design a simple task that could objectively assess detail visibility, it is worth noting that people were likely able to take into account the surrounding context of the patches to aid in their judgment. Indeed, all participants reported using some kind of continuation of lines or gradient of light and dark to make their judgment. Objective assessment has strong value in helping us better understand how people perceive visual content and potentially interact with it. However, particularly with natural images, it can be challenging to isolate the information of interest that people use to complete a task. Future work could explore developing a standard contrast perception task for natural scenes with a larger sample of users and image content, perhaps by embedding standardized targets as in Sebastian et al. [33]. Furthermore, since dichoptic tone mapping methods are readily implementable in near-eye stereoscopic displays, it would be useful to further explore performance-based measures of tasks relevant for VR applications that incorporate dynamic content and stereoscopic viewing.

## 6 CONCLUSION

Despite many advances in imaging devices, display technology, and computational algorithms, digital scene reproduction remains a challenging process. With the rise of more commonplace stereoscopic displays for VR applications, it is appealing to consider how binocular vision can be leveraged to improve or augment existing reproduction pipelines beyond conventional stereoscopic imaging. Future work on developing dichoptic methods may benefit from examining what differs between scenes that elicit more or less improvement in subjective image quality and contrast, as well as focusing on gaining a better understanding of how these methods affect and enhance 3D impressions.

## Supplementary Material

Supplementary Material

## Figures and Tables

**Table 1.** Overview of Experiments

Exp.	Task	Criterion	Participants	Scenes
1	rating	image quality	16*	18
2	rating	detail visibility	16*	18
3	rating	3D impression	16	18
4	2AFC	image quality	16	18
5	2AFC	detail visibility	16	18
6	performance	detail visibility	24	8

Asterisk indicates that the same 16 people participated in Experiments 1 and 2.

**Table 2.** Summary of Dichoptic and Non-dichoptic Conditions in Experiments

Dichoptic Conditions	Non-Dichoptic Conditions
Zhang et al. [41]	average tone map global tone map local tone map
Dichoptic exposure	proper exposure high exposure low exposure
Zhong et al. [42] (DiCE)	C1 (low-light detail) C2 (highlight detail)

**Table 3.** Results of Omnibus Statistical Tests of the Ratings in Experiments 1, 2, and 3

Tone Map			Exposure		
Overall Preference					
*χ* ^*2*^	*p*	*W*	*χ* ^*2*^	*p*	*W*
34.10	<0.001*	0.71	27.00	<0.001*	0.56
Detail Visibility					
*χ* ^*2*^	*p*	*W*	*χ* ^*2*^	*p*	*W*
34.91	<0.001*	0.73	37.81	<0.001*	0.79
3D Impression					
*χ* ^*2*^	*p*	*W*	*χ* ^*2*^	*p*	*W*
34.74	<0.001*	0.72	29.83	<0.001*	0.62

For each dichoptic method and task, we report the *χ*^2^ statistic and p-values from Friedman tests, along with Kendall’s coefficient of concordance for the participants (W). Statistically significant comparisons with a p-value threshold of 0.05 are marked with an asterisk.

**Table 4.** Results of Follow-up Statistical Tests for the Participant Ratings in Experiments 1, 2, and 3

Tone Map			Exposure		
Overall Preference					
	*z-Stat*	*p*		*z-Stat*	*p*
Average	−1.52	0.13	Proper	0.90	0.37
Global	−3.01	0.003*	High	−2.60	0.01*
Local	2.37	0.02*	Low	2.01	0.04
Detail Visibility					
	*z-Stat*	*p*		*z-Stat*	*p*
Average	−1.89	0.06	Proper	−1.32	0.19
Global	−3.21	0.001*	High	−3.53	<0.001*
Local	2.12	0.03	Low	1.68	0.09
3D Impression					
	*z-Stat*	*p*		*z-Stat*	*p*
Average	−3.45	<0.001*	Proper	−3.09	0.002*
Global	−3.44	<0.001*	High	−3.33	<0.001*
Local	−3.07	0.002*	Low	−2.97	0.003*

Wilcoxon signed-rank tests were run comparing the ratings of each dichoptic condition against its three non-dichoptic comparisons, using z-statistics to assess significantly different ratings with a p-value threshold of 0.05. Each row indicates the results for comparing one dichoptic condition (tone map or exposure) with the listed non-dichoptic conditions. We corrected for multiple comparisons using a false discovery rate (fdr) of 0.05 [2]. Comparisons for which the ratings were significantly different following fdr correction are marked with an asterisk.

**Table 5.** Summary of Conditions that are Compared in the 2AFC Tasks in Experiments 4 and 5

Dichoptic	vs.	Non-Dichoptic
dichoptic tone map [41]	vs.	local tone map
dichoptic exposure	vs.	low exposure
DiCE [42]	vs.	C1 (low-light detail)
DiCE [42]	vs.	C2 (highlight detail)

**Table 6.** Results of Statistical Tests for 2AFC Task in Experiments 4 and 5

Preference				
*Non-Dichoptic*	*Mean*	*Standard Deviation*	*t-Stat*	*p*
Local	0.23	0.13	−7.99	<0.001*
Low	0.29	0.13	−6.35	<0.001*
C1	0.55	0.17	1.16	0.26
C2	0.46	0.16	−1.07	0.3
Detail Visibility				
*Non-Dichoptic*	*Mean*	*Standard Deviation*	*t-Stat*	*p*
Local	0.17	0.10	−12.73	<0.001*
Low	0.24	0.14	−7.17	<0.001*
C1	0.49	0.23	−0.19	0.85
C2	0.41	0.20	−1.66	0.12

For each dichoptic to non-dichoptic comparison, a two-tailed, single sample t-test was conducted, with the null hypothesis that the mean proportion was equal to chance (0.5) and a significance threshold of p < 0.05. Comparisons for which the mean was significantly different from 0.5 are marked with an asterisk.

**Table 7.** Results of Statistical Tests for DiCE Scene-based Analysis Shown in Figure 6 Panels C & D

DiCE vs. C1			DiCE vs. C2		
*σ*	*r [95% CI]*	*p*	*σ*	*r [95% CI]*	*p*
1	0.70 [0.35 0.88]	0.001*	1	−0.67 [−0.87 −0.29]	0.002*
1.5	0.71 [0.36 0.88]	0.001*	1.5	−0.69 [−0.87 −0.33]	0.002*
3	0.71 [0.36 0.88]	0.001*	3	−0.70 [−0.88 −0.35]	0.001*
C2- vs. C1-preferred visual detail					
*σ*	*t*		*p*		
1	−4.26		0.001*		
1.5	−4.13		0.001*		
3	−3.97		0.001*		

Top: For each filter size (*σ* in pixels), the correlation coefficient with 2AFC responses (r), 95% confidence intervals (CI), and p values are reported. Significant correlations (p < 0.05) are marked with an asterisk. Bottom: For each filter size, a two-tailed, un-paired t-test was conducted to compare C1-preferred scenes and C2-preferred scenes, with the null hypothesis that the mean local detail difference between the two scene categories were not different from each other and a significance threshold of p < 0.05. Comparisons for which the means were significantly different between C1- and C2-preferred scenes are marked with an asterisk.

**Table 8.** Results of Logistic Regression Model Fit to Data from Experiment 6

Tone Map Conditions			
*Condition*	*Coefficient Estimate*	*Odds Ratio*	*p*
Average	0.11	1.12	0.25
Global	−0.18	0.83	0.06
Local	0.10	1.11	0.32
*Random Effects*	*Standard Deviation*	*Lower Bound*	*Upper Bound*
Scene	0.23	0.11	0.51
Participant	0.51	0.35	0.75
Exposure Conditions			
*Condition*	*Coefficient Estimate*	*Odds Ratio*	*p*
Proper	−0.04	0.96	0.68
High	−0.02	0.98	0.88
Low	−0.12	0.89	0.22
*Random Effects*	*Standard Deviation*	*Lower Bound*	*Upper Bound*
Scene	0.35	0.19	0.65
Participant	0.52	0.35	0.76

For each fixed effect (conditions), coefficients and odds ratios reflect the change in probability of correct responses associated with each non-dichoptic condition relative to its dichoptic comparison. No coefficients were statistically significant at the threshold of p < 0.05. Contributions of scene and participant were modeled as random effects. There was moderate variability among different scenes and high variability among participants as shown by the standard deviations, which indicates how much each participant or scene deviates from the estimated average effects of the conditions.

## CCS Concepts:

- **Computing methodologies** → **Perception**; *Image processing*; *Computational photography*; Virtual reality;
