# Supplementary material for: A Re-examination of Dichoptic Tone Mapping
Source: ACM Trans Graph. Author manuscript; Available in PMC 2022 Apr 12. (PMC9004687; doi:10.1145/3443702)
Supplement: Supplementary Material [file NIHMS1787720-supplement-Supplementary_Material.pdf]

Supplementary Material for: A Re-examination of Dichoptic Tone Mapping

MINQI WANG and EMILY A. COOPER, University of California, Berkeley, USA

In this supplementary document, we present additional analyses of the data from the user studies. We examine whether individual differences in ratings were related to the participant's eye dominance (Experiments 1, 2, and 3). We also report Differential Mean Opinion Scores (DMOS) for these data, separated out by scene. We report a resampling analysis of the 2AFC data examining consistently of results across subsets of scenes (Experiments 4 and 5). Finally, we report an analysis of the effect of stimulus contrast on objective task performance (Experiment 6).

1 BINOCULAR RIVALRY AND EYE DOMINANCE (EXPERIMENTS 1, 2, & 3)

When presented with different stimuli to the two eyes, the visual system needs to reconcile these stimuli to form a single percept. For dichoptic tone mapping to work as intended, the visual system should merge the two eyes' images in a way that maximizes information, regardless of channel that the information came in (e.g., left or right eye). However, people can have a perceptually dominant eye, such that the image shown to that eye is favored or contributes more to their percept. We wanted to know whether there were any differences in people's subjective ratings of dichoptic conditions that can be attributed to eye dominance. To do so, we conducted a *sensory eye dominance* test based on binocular rivalry for each of the participants in Experiments 1–3. We performed post hoc analyses to ask whether the participants' eye dominance status might be predictive of their rating results.

1.1 Stimuli and Procedure

Two orthogonal sine wave gratings were presented to the eyes simultaneously via the haploscope (as illustrated in Figure 1). This is a standard stimulus for binocular rivalry tests [1]. Participants were instructed to press down buttons continuously to indicate whether they saw the grating predominately tilted top left or top right, and to not press any key if they saw an equal mixture of both orientations. The procedure took about three minutes. Participants had a short one-minute practice before the actual test.

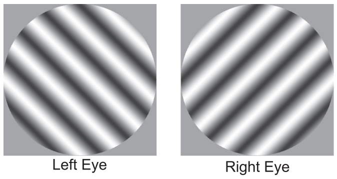

Fig. 1. Stimuli used during rivalry test.

© 2021 Association for Computing Machinery.
0730-0301/2021/04-ART13 $15.00
https://doi.org/10.1145/3443702

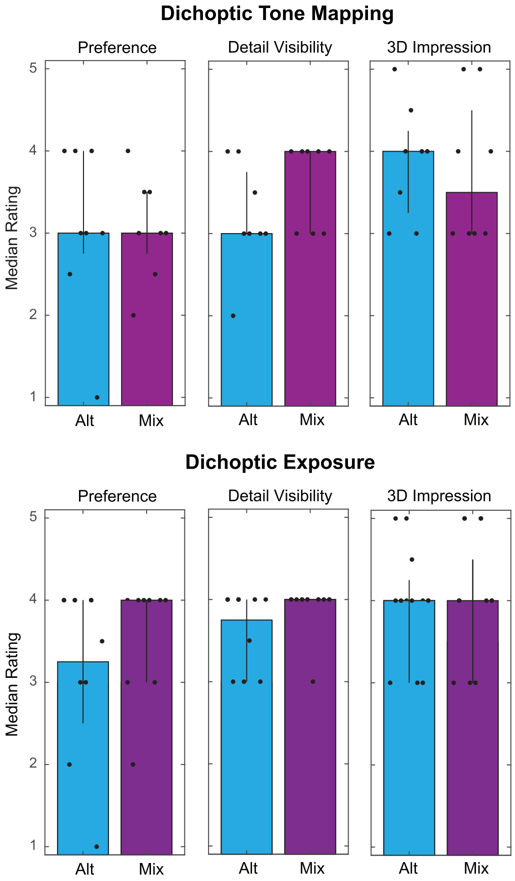

Fig. 2. Results of post hoc analysis of the relationship between eye dominance and median ratings. For each panel, the dichoptic condition is split up between participants who were defined as "Alternators" (Alt) and "Mixers" (Mix). The top row shows the results for the three ratings tasks for the dichoptic tone mapping condition, the bottom row shows the same results for the dichoptic exposure condition. The black dots indicate individual participants, the bars indicate the median across participants, and the black lines indicate the 25th and 75th percentiles.

1.2 Results

The dominant eye of each participant was determined by calculating the proportion of time that the participant reported seeing the grating presented to each eye. We also calculated the proportion of time each participant saw a mixture of both eyes' stimuli. We categorized each participant as a "Mixer" if their proportion of time seeing a mixture was greater than the median across all participants (52 seconds), or an "Alternator" if their proportion of time seeing a mixture was less than than the median. The rating results for the three experiments are replotted in Figure 2, with

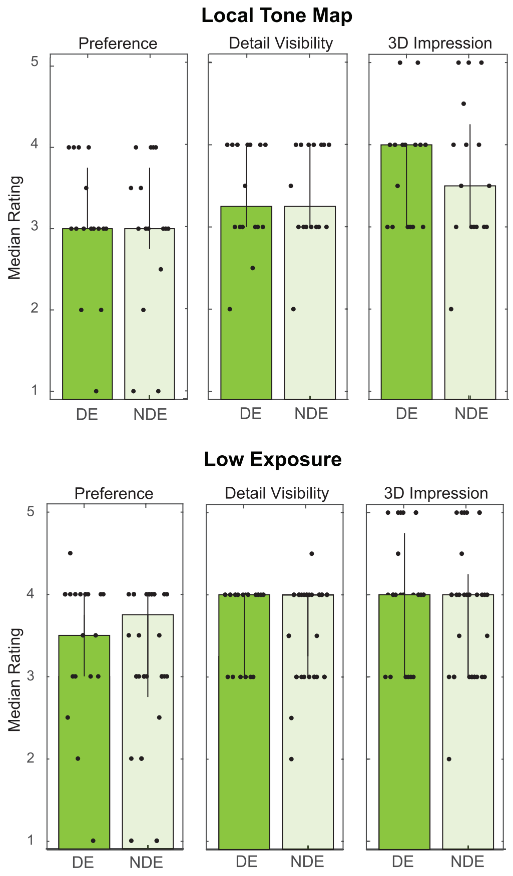

Fig. 3. Results of post hoc analysis of which eye saw the higher rated component image (i.e., the local tone map or the low exposure) during dichoptic conditions. For each panel, the median ratings of a dichoptic condition are shown, split into trials for which the higher rated component image was seen by dominant eye (DE) or non-dominant eye (NDE). The top row shows the results for the dicophtic tone map condition, the bottom row shows the same results for the dichoptic exposure condition.

Table 1. Results of Wilcoxon Statistical Tests Examining Differences between the Responses of Participants Who Were Alternators and Mixers (Top) and Differences between Responses When the Preferred Component Image was Presented to the Dominant versus Non-dominant Eye (Bottom)

Tone Map			Exposure		
Alternator vs. Mixer Rating					
	z-Stat	*p*		*z-Stat*	*p*
Preference	0.22	0.83	Preference	−0.85	0.40
Detail	−1.32	0.19	Detail	−1.42	0.16
3D	0.38	0.70	3D	−0.23	0.82
Dominant vs. Non-Dominant Eye Rating					
	z-Stat	*p*		*z-Stat*	*p*
Preference	0.00	1.00	Preference	0.28	0.78
Detail	−0.28	0.78	Detail	−0.52	0.60
3D	0.32	0.75	3D	0.38	0.71

We used z-statistics to assess significantly different ratings with a p-value threshold of 0.05. Each row indicates the results for a particular perceptual quality. For unpaired samples (top), the rank sum test was used. For paired samples (bottom), the signed-rank test was used.

data shown separately for Alternators and Mixers. There were no statistically significant differences between Alternator's and Mixer's ratings on dichoptic viewing conditions across all experiments (Table 1, upper panel). Descriptively, Mixers tended to rate the dichoptic tone map condition higher for detail visibility, and the dichoptic exposure condition higher for preference, but these differences were not statistically significant.

Since the dichoptic trials were repeated with the component images switched between the two eyes, we also explored if there was a difference in whether the higher-rated component image in the dichoptic pair was seen by the dominant eye or the non-dominant eye. User ratings tended to be similar but not identical between these two repeats (average Spearman correlation of 0.66, 0.70, and 0.58 for Experiments 1, 2, and 3, respectively). Some of this variability may be due to inconsistent preferences, but some may be due to the different images seen by the dominant eye. The results of this analysis are plotted in Figure 3, and statistical comparisons are reported in Table 1 (lower panel). We found no consistent or statistically significant improvements when the higher-rated component image was seen by the dominant eye.

In summary, we did not find evidence for a relationship between the person's sensory eye dominance and their responses in our user studies. This may indicate that the perceptual outcome is driven more by the content of the stimuli (i.e., the better component image) than by which eye sees which stimulus. This result is in line with results from Yang et al., in which they switched the presentation of the dichoptic pair for each eye and found no difference in 2AFC responses [4]. The DiCE study also included a supplementary study on eye dominance in which they tested *sighting* eye dominance and found no systematic relationship with contrast enhancement [5]. It is important to note that the existing analyses of eye dominance effects, including our own, have used relatively small sample sizes for analyses of individual differences. As such, there may still be robust individual differences in binocular combination that contribute to the variability of preferences for dichoptic methods, which may be explored in future work.

2 DIFFERENTIAL MEAN OPINION SCORES (EXPERIMENTS 1, 2, & 3)

Figures 4–6 show a differential mean opinion score (DMOS) analysis for each scene included in Experiments 1–3 [2]. DMOS scores were calculated by taking the difference between the rating in a reference condition (in this case, dichoptic tone map or dichoptic exposure) with the rating in a test condition (non-dichoptic) for each scene, participant, and repeat. We then averaged scores across participants for each condition on a scene-by-scene basis. Each figure panel shows the results for one scene (tone mapping conditions in green and exposure conditions in blue), with the mean and the 95% confidence interval of the DMOS indicated. Values greater than zero indicate that the dichoptic condition was preferred, and values less than zero indicate that the non-dichoptic condition was preferred. These results are consistent with the raw ratings analysis: For Experiments 1 and 2 (preference and detail visibility), we see that one of the non-dichoptic conditions is

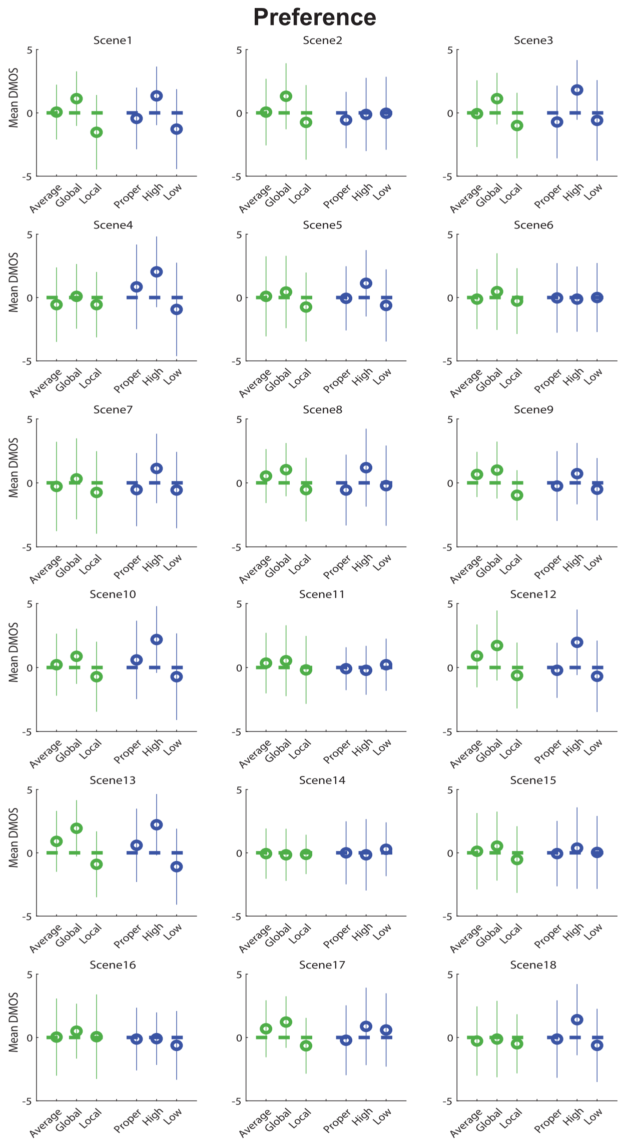

Fig. 4. Results of Experiment 1 converted to differential mean opinion scores. Green circles indicate tone map conditions, and blue circles indicate exposure conditions with vertical lines showing the 95% confidence interval.

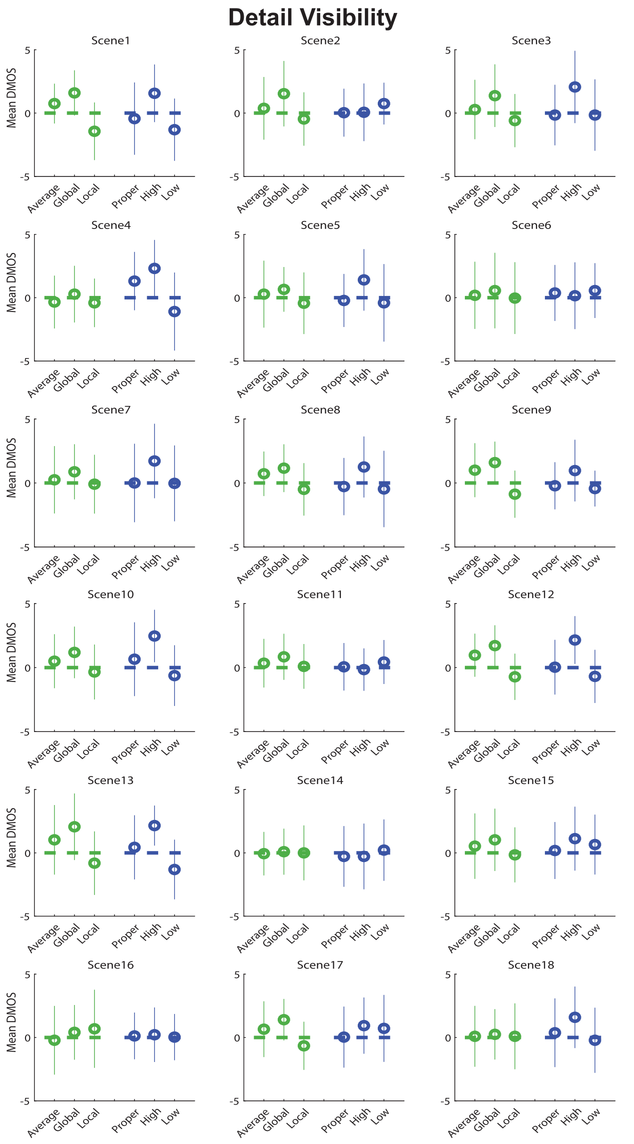

Fig. 5. Results for Experiment 2 plotted in the same manner as Figure 4.

often rated higher than the dichoptic reference (data points below zero). For Experiment 3 (3D impression), we see that the dichoptic condition is almost always rated higher than the non-dichoptic conditions (data points above zero).

3 SCENE SAMPLE ANALYSIS (EXPERIMENTS 4 & 5)

It is important to consider whether our user study results could be specific to the selected scenes, rather than a generalizable observation about dichoptic tone mapping. This is a challenging question to answer, but we can gain some insight by examining the variability within the data we have. To examine the relationship between the number of natural images tested and the conclusions drawn from the user studies, we performed a post hoc resampling analysis. We asked how variable the results for Experiments 4 and 5 would be for a range of smaller image samples from 3 images to 15 images. Our logic was that, for a given sample size, a large amount of variability suggests that conclusions from a given study would be less generalizable. Thus, we repeatedly sampled our user response data from a subset of the original 18 scenes randomly and recalculated the average proportion dichoptic chosen. For example, for a simulation using 3 scenes, we might randomly choose Scenes 1, 2, and 3 in the first simulation, but Scenes 2, 3, and 10 might be chosen for the next simulation. We performed each subsampling (3, 6, 9, 12, and 15 scenes) for 100 simulations to obtain the average and standard deviation. The results are shown in Figure 7. As expected, lower numbers of scenes result in more variability across simulations. For the local tone map and low exposure comparison conditions, the bulk of the results showed the non-dichoptic images being consistently preferred even for very small

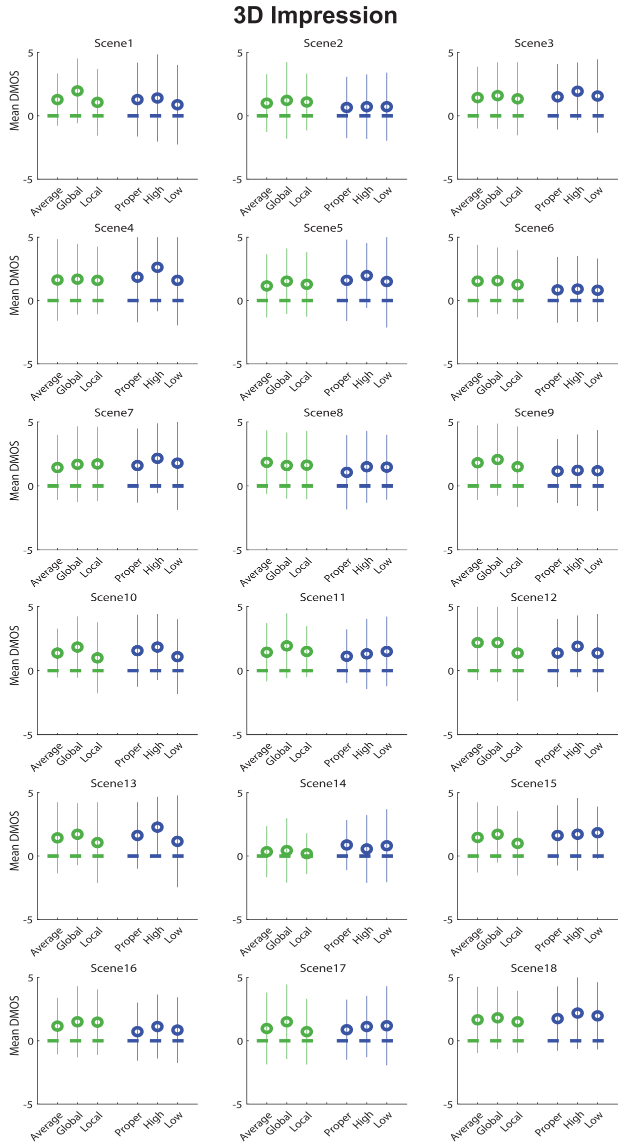

Fig. 6. Results for Experiment 3 plotted in the same manner as Figure 4.

sample sizes. For the DiCE comparison conditions, small sample sizes could result in consistent above or below chance preferences for the dichoptic tone maps. But once at least 10 images were included, the range of results was highly consistent with the results with the full 18 image set. While this technique only allows us to sample the variability within the current images, it provides some support to the notion that the conclusions are not specific to particular images.

4 EFFECT OF CONTRAST ON OBJECTIVE TASK PERFORMANCE (EXPERIMENT 6)

We conducted a post hoc analysis of the results from Experiment 6 to examine whether patch contrast was predictive of task performance. This analysis focused on the trials with non-dichoptic presentation, because on these trials we have a reasonable model

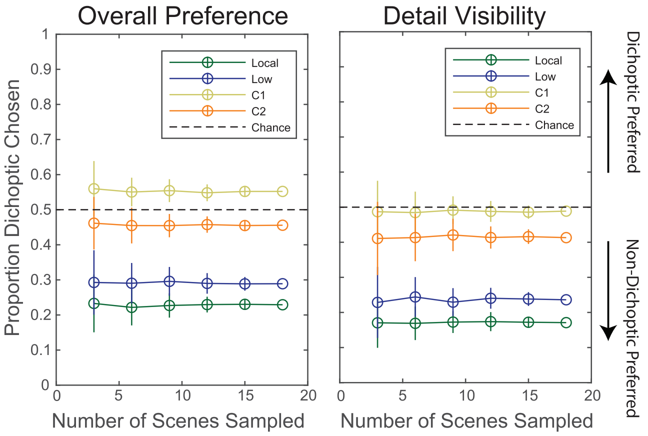

Fig. 7. Results for simulating Experiments 4 and 5 using the data from a subset of scenes. Each line represents one condition's mean, and vertical bars indicate +/- one standard deviation. The x-axis indicates how many scenes were randomly sampled, equally spaced from 3 to the actual number of scenes that we tested, which was 18.

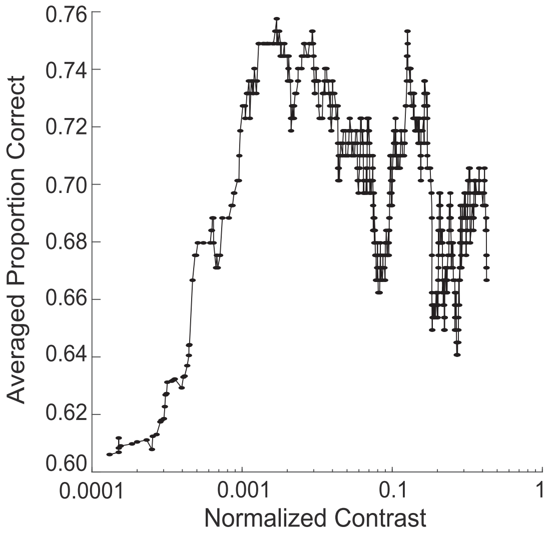

Fig. 8. Analysis of the effect of stimulus contrast on the objective task performance in Experiment 6. The x-axis is the normalized contrast on a log scale, and the y-axis is the average performance. Data were smoothed with a moving average filter with a span of 77 samples. Edge cases that exceeded the half-width of the filter were cropped.

for perceived contrast that does not rely on assumptions about binocular combination. For each unique stimulus presented with a non-dichoptic tone map (n = 768), we computed the normalized contrast of the patch by dividing the standard deviation of pixel gray-scale values by the mean gray-scale value. This approach calculates contrast in units that are approximately proportionate to the overall brightness of the patch, reflecting Weber's Law for contrast detection [3]. Due to the limited number of user responses available to compute accuracy for each individual stimulus (in the Latin Square design, a given patch and tone map combination was only seen by three users), we used a sliding window (of size n/10)

to compute the average proportion correct responses as a function of the normalized contrast. The results are plotted in Figure 8. Note that the contrast values on the abscissa are plotted on a log scale. At very low contrasts, the users' performance was positively related to contrast, with average performance increasing rapidly by ~10% as contrast increased above the minimum. However, above a certain normalized contrast level (≈0.002), the performance no longer increased systematically with contrast. These results suggest that visible contrast is important for enabling performance on the task, but that above a relatively low threshold the contrast becomes sufficient and no longer limits performance.

REFERENCES

[1] Chao Han, Zijiang J. He, and Teng Leng Ooi. 2018. On sensory eye dominance revealed by binocular integrative and binocular competitive stimuli. *Investig. Ophthalm. Vis. Sci.* 59 (2018), 5140–5148.

[2] Rafal Mantiuk, Anna Lewandowska, and Radoslaw Mantiuk. 2012. Comparison of four subjective methods for image quality assessment. *Comput. Graph. Forum* 31, 8 (2012), 2478–2491.

[3] Anasuya S. Patel and Ronald William Jones. 1968. Increment and decrement visual thresholds. *J. Optic. Soc. Amer.* 58 5 (1968), 696–9.

[4] Xuan S. Yang, Linling Zhang, Tien-Tsin Wong, and Pheng-Ann Heng. 2012. Binocular tone mapping. *ACM Trans. Graph.* 31, 4 (2012).

[5] Fangcheng Zhong, George Alex Koulieris, George Drettakis, Martin S. Banks, Mathieu Chambe, Frédo Durand, and Rafał K. Mantiuk. 2019. DiCE: Dichoptic contrast enhancement for VR and stereo displays. *ACM Trans. Graph.* 38, 6 (2019).
